# Single Cell Transcriptome Data Analysis Defines the Heterogeneity of Peripheral Nerve Cells in Homeostasis and Regeneration

**DOI:** 10.3389/fncel.2021.624826

**Published:** 2021-03-22

**Authors:** Bing Chen, Matthew C. Banton, Lolita Singh, David B. Parkinson, Xin-peng Dun

**Affiliations:** ^1^Department of Neurology, The Affiliated Huai'an No. 1 People's Hospital of Nanjing Medical University, Huai'an, China; ^2^Faculty of Health, School of Biomedical Science, University of Plymouth, Plymouth, United Kingdom; ^3^Faculty of Health, Peninsula Medical School, University of Plymouth, Plymouth, United Kingdom; ^4^School of Pharmacy, Hubei University of Science and Technology, Xianning, China

**Keywords:** peripheral nerve, injury, cell type identification, marker genes, cell-cell communication, scRNA-seq analysis

## Abstract

The advances in single-cell RNA sequencing technologies and the development of bioinformatics pipelines enable us to more accurately define the heterogeneity of cell types in a selected tissue. In this report, we re-analyzed recently published single-cell RNA sequencing data sets and provide a rationale to redefine the heterogeneity of cells in both intact and injured mouse peripheral nerves. Our analysis showed that, in both intact and injured peripheral nerves, cells could be functionally classified into four categories: Schwann cells, nerve fibroblasts, immune cells, and cells associated with blood vessels. Nerve fibroblasts could be sub-clustered into epineurial, perineurial, and endoneurial fibroblasts. Identified immune cell clusters include macrophages, mast cells, natural killer cells, T and B lymphocytes as well as an unreported cluster of neutrophils. Cells associated with blood vessels include endothelial cells, vascular smooth muscle cells, and pericytes. We show that endothelial cells in the intact mouse sciatic nerve have three sub-types: epineurial, endoneurial, and lymphatic endothelial cells. Analysis of cell type-specific gene changes revealed that Schwann cells and endoneurial fibroblasts are the two most important cell types promoting peripheral nerve regeneration. Analysis of communication between these cells identified potential signals for early blood vessel regeneration, neutrophil recruitment of macrophages, and macrophages activating Schwann cells. Through this analysis, we also report appropriate marker genes for future single cell transcriptome data analysis to identify cell types in intact and injured peripheral nerves. The findings from our analysis could facilitate a better understanding of cell biology of peripheral nerves in homeostasis, regeneration, and disease.

## Introduction

Our nervous system comprises of two parts: the central nervous system (CNS) and the peripheral nervous systems (PNS). The CNS is the part of the nervous system consisting primarily of the brain and spinal cord. The brain is encased in the skull and the spinal cord is protected by the vertebrae. Outside of the brain and spinal cord is the PNS, which due to the lack of protection, is prone to damage from traumatic injuries. It is not until the PNS is damaged that the huge consequences become clear. These injuries often lead to the development of neuropathic pain and life-long loss of both motor and sensory function. The injuries not only greatly compromise the quality of life of affected individuals but also impose a great financial burden on the healthcare system (Deumens et al., 2010).

In contrast to CNS injury, the PNS has a stunning ability to regenerate following injury. This remarkable regenerative ability is achieved by the rapid activation of an intrinsic regeneration program in damaged neurons and through a permissive environment created by supporting cells in the distal nerve stump such as Schwann cells, nerve fibroblasts, endothelial cells and infiltrated immune cells (Chandran et al., 2016; Jessen and Mirsky, 2016; Renthal et al., 2020). Thus, identifying all the cell types in a healthy peripheral nerve and studying the cell type changes following injury could facilitate our understanding of key cellular and molecular mechanisms regulating peripheral nerve regeneration. Previous studies frequently use methods such as immunohistochemistry, *in situ* hybridization, electron microscopy and transgenic mice expressing fluorescent proteins to identify cell types in the peripheral nerves (Mallon et al., 2002; Stierli et al., 2018; Ydens et al., 2020). However, usually a combination of these approaches are required in order to identify most of the cell types present, and cells with low abundance are much harder to identify with these techniques (Stierli et al., 2018).

The advance of single-cell RNA sequencing (scRNA-seq) technologies and the development of bioinformatics pipelines not only enable us to define the heterogeneity of cell types in a selected tissue but also allow us to study a cell-specific gene expression profile (Chen et al., 2019b). Single-cell RNA sequencing technologies have been widely used in different research fields to reveal complex and rare cell populations, to track the trajectories of distinct cell lineages, and to study the gene expression profiles of selected cell types (Hwang et al., 2018). However, this technique has only recently been applied to study the cell types and gene expression profiles of intact and injured mouse peripheral nerves (Carr et al., 2019; Toma et al., 2020; Wolbert et al., 2020). In this report, we re-analyzed recently published single-cell RNA sequencing data sets and provide our rationale to define the heterogeneity of cells in intact and injured peripheral nerves. We compared the changes of cell type composition and gene expression patterns between intact and injured sciatic nerve with our analysis, and revealed cell-cell communications in intact and injured sciatic nerve. We also provide suggested markers for future single cell transcriptome data analysis for the identification of cell types in intact and injured peripheral nerves. The findings from our analysis will, we hope, facilitate a better understanding of peripheral nerve cell biology in homeostasis, regeneration and disease.

## Methods

### Computational Analysis of Single-Cell RNA Sequencing Data Sets

scRNA-seq data set GSE142541 for intact mouse sciatic nerve and the brachial nerve plexus (Wolbert et al., 2020), data set GSE147285 for intact mouse sciatic nerve and post-injury day 3 distal nerve (Toma et al., 2020), and data set GSE120678 for post-injury day 9 distal nerve (Carr et al., 2019) were downloaded from the NCBI GEO database. Data sets were analyzed using the Seurat v.3.2.1 (https://satijalab.org/seurat/) and sctransform v.0.3 R packages using R v.4.0.2. Quality control plots of number of features, counts and percentage mitochondrial content per cell were plotted for each data set and used to determine filtering conditions. For the quality control of intact mouse sciatic nerve data set GSE42541, cells were filtered using the following conditions: number of features per cell 200–2,000 and percent mitochondrial DNA content per cell <8%. For the quality control of intact mouse sciatic nerve data set GSE147285, filtering conditions were: number of features per cell 200–6,000 and percent mitochondrial DNA content per cell <8%. For the quality control of post-injury day 3 nerve data set, cells were filtered using the following conditions: number of features per cell 200–6,000 and percent mitochondrial DNA content per cell <6%. For the quality control of post-injury day 9 nerve data set, cells were filtered using the following conditions: number of features per cell 200–4,000 and percent mitochondrial DNA content per cell <8%.

Filtered cell data were normalized, variable genes identified and data scaled using SCTransform, a recently published highly effective method for removing technical artifacts from scRNAseq data while retaining biological heterogeneity (Hafemeister and Satija, 2019). The dimensionality of the dataset was determined using elbow plots to identify the appropriate number of principal components used for clustering. Cell clustering was performed using the FindNeighbors and FindCluster functions in Seurat. Differentially expressed genes (DEG) were identified using the FindAllMarkers Seurat function using the Wilcoxon rank sum test for genes with a minimum 0.25 log fold change between clusters and expressed in at least 10% of cells between clusters, unless otherwise stated. To annotate the clusters, genes differentially expressed in a one vs. all cluster comparison were queried for known expression in a literature search and gene expression plotted. Integration of data sets was performed using Seurat (v.3.2.1) using the SCTransform normalized data and PrepSCTIntegration function. DEG between conditions within each cluster were identified using the FindMarkers function using the same argument values as the FindAllMarkers Seurat function described above. Cell clustering was visualized using t-distributed stochastic neighbor embedding (tSNE) using the FeaturePlot function in Seurat. t-SNE gene expression overlays, violin plots, dot plots and heatmaps for cell type specific marker genes were also plotted using Seurat specific functions.

### Marker Genes for the Identification of Cell Clusters

Cell clusters were identified based on the use of the following established marker genes for cell types of mouse sciatic nerves (Evrard et al., 2018; Zhao et al., 2018; Carr et al., 2019; Renthal et al., 2020; Toma et al., 2020; Wolbert et al., 2020; Xie et al., 2020; Ydens et al., 2020). Egfl7, Ecscr, Pecam1/Cd31, Tie1, Emcn, Cdh5, and Esam for endothelial cells. Sox17, Spock2, and Rgcc for epineurial endothelial cells, Lrg1 and Icam1 for endoneurial endothelial cells. Lyve1, Mmrn1, Flt4, and Prox1 for lymphatic endothelial cells. Des, Tpm2, Myh11, Acta2, Mylk, Myom1, and Myocd for vascular smooth muscle (VSM) cells. Rgs5, Kcnj8, and Pdgfrb for pericytes. Sox10, Plp1, and S100b for Schwann cells. Cdh2 and L1cam for non-myelinating Schwann cells. Mbp, Mpz, Mag, and Egr2 for myelinating Schwann cells. Dcn, Mfap5, Serpinf1, and Gsn for fibroblasts. Sfrp2, Dpt, Pcolce2, Adamts5, Pi16, Sfrp4, Prrx1, Comp, and Ly6c1 for epineurial fibroblasts. Cldn1/claudin-1, Lypd2, Ntn4, Msln, Ntng1, Slc2a1/Glut1, and Mpzl2 for perineurial cells. Sox9, Osr2, Wif1, Abca9, Cdkn2a, Cdkn2b, and Plxdc1 for endoneurial fibroblasts. Dlk1, Mest, Cilp, Tnc, Plagl1, and Ptn for differentiating fibroblasts. Pdgfra, Thy1, and Cd34 for mesenchymal cells. Ptprc/CD45 and Cd52 as general marker for immune cells. Aif1/Iba1, Cd68, Mrc1/Cd206, and Adgre1/F4/80 for macrophages. Retnla and Clec10a for epineurial macrophages. Ccl6, Fcgr3, Cx3cr1, Csf1r, Cd300a, and Clec4e for monocytes. S100a8, S100a9, Cxcr2, and Cxcl2 for neutrophils. Cma1, Mcpt4, Mcpt1, and Kit for mast cells. Cd3g, Cxcr6, Trac, and Cd3e for T cells. Nkg7, Klrk1, and Ncr1 for natural killer (NK) cells. Bank1, Cbfa2t3, Taok3, Ms4a1, Cd19, and Cd79a for B cells (Evrard et al., 2018; Zhao et al., 2018; Carr et al., 2019; Renthal et al., 2020; Toma et al., 2020; Wolbert et al., 2020; Xie et al., 2020; Ydens et al., 2020).

### Peripheral Nerve Surgery

Two-month-old C57BL/6J and PLP-GFP mice were used in the study, Schwann cells are labeled with GFP in PLP-GFP mice (Mallon et al., 2002; Dun et al., 2019). All work involving animals was carried out according to Home Office regulation under the UK Animals Scientific Procedures Act 1986. Ethical approval for all experiments was granted by Plymouth University Animal Welfare and Ethical Review Board. Mice were housed in a controlled laboratory environment (temperature 22 ± 2°C, humidity 50–60%, 12-h light/dark cycle), and fed with standard rodent diet and water added *ad libitum*. For sciatic nerve transection injury, six male mice were anesthetized with isoflurane, the right sciatic nerve was exposed and transected at approximately 0.5 cm proximal to the nerve trifurcation site and no re-anastamosis of the severed nerve was performed. Overlying muscle was sutured and the skin was closed with an Autoclip applier. Mice undergoing surgery were given appropriate post-operative analgesia, 0.05% bupivacaine solution, topically applied above the muscle suture before applying surgical clips. Meloxicam (5 mg/kg) injections were given just before recovery from anesthetic. Mice undergoing surgery were given nesting material and cage enrichment to minimize the risk of autotomy. Animals under surgery were monitored daily. At day 3 and day 7 post surgery, animals were euthanased humanely using carbon dioxide in accordance with UK Home Office regulations.

### Edu Labeling and Cell Proliferation Assay

Proliferation was measured using the Click-iT™ EdU kit (Thermo Fisher Scientific). At 7 days post injury, a stock solution containing 2 mg of EdU (Invitrogen; cat.no C1033) was administered to each mouse via intraperitoneal injection (total volume 200 μL in PBS) and mice were sacrificed 3 h later. Sciatic nerves were collected and fixed in 4% paraformaldehyde/PBS overnight. Next day, nerves were washed 3 times with PBS and then cryopreserved in 30% sucrose/PBS overnight. Subsequently, nerves were embedded in OCT medium and sectioned longitudinally on a cryostat at a thickness of 10 μm. Sections were permeabilised with 0.25% Triton X-100 plus 1% bovine serum albumin (BSA) in PBS for 45 min and then blocked with blocking buffer (3% BSA plus 0.05% Triton X-100 in PBS) for 1 h at room temperature. Sections of the sciatic nerve were then incubated with the EdU Click-iT reaction cocktail (Invitrogen; cat.no C1033) for 30 min at room temperature followed by three PBS washing steps for 10 min each. Sections or cells were incubated with S100 primary antibodies (1:500 diluted in blocking buffer) overnight at 4°C. The next day, sections were washed with PBS (3 × 10 min) and then incubated with secondary antibody plus Hoechst dye (1:500 diluted in blocking buffer) for 1 h at room temperature. Finally, sections were washed with PBS (3 × 10 min) and mounted with Citifluor (Agar Scientific, R1320) for imaging with a LeicaSPE confocal microscope.

### Immunohistochemistry

Sciatic nerve were dissected out and fixed 5 h in 4% paraformaldehyde (in PBS, PH7.2) at 4°C. Nerves were then washed in PBS (3 × 10 min) and dehydrated in 30% sucrose (in PBS) overnight at 4°C. Subsequently, nerves were embedded in OCT medium and sectioned on a cryostat at a thickness of 12 μm. Sections were permeabilised with 0.25% Triton X-100 plus 1% bovine serum albumin (BSA) in PBS for 45 min and then blocked with blocking buffer (3% BSA plus 0.05% Triton X-100 in PBS) for 1 h at room temperature. Sections were incubated with Ki67 (Abcam, ab15580), S100 (Dako, Z0311), Csf3r (Thermofisher, BS-2574R), Spock2 (Thermofisher, BS-11966R), Rgcc (Thermofisher, BS-9079R), Lrg1 (Invitrogen, PA5-96832) primary antibody (1:100 diluted in blocking buffer) overnight at 4°C. The next day, sections were washed with PBS (3 × 10 min) and then incubated with donkey anti-rabbit secondary antibody conjugated with Alexa Fluor 488 or 568 (1:300 diluted in blocking buffer) for 1 h at room temperature. Hoechst dye (1:500) was also added into secondary antibody solution in order to label cell nuclei. Finally, sections were washed with PBS (3 × 10 min) and mounted with Citifluor (Agar Scientific, R1320) for imaging with a LeicaSPE confocal microscope.

### Identifying Ligand-Receptor Interactions Between the Cell Clusters in Intact and Injured Nerves

Cell-cell communication between Seurat identified clusters was analyzed using CellPhoneDB (https://www.cellphonedb.org) (Efremova et al., 2020) using the SCTransform normalized gene counts. Mouse HGNC symbols for genes present in the CellPhoneDB database were converted to human orthologs using the R package biobtreeR (Gur, 2019). CellPhoneDB was run using 1,000 statistical iterations and a threshold of at least 10% of cells expressing a gene in a cluster was used.

## Results

### Computational Analysis of scRNA-seq Data Sets

**Figure 1:**
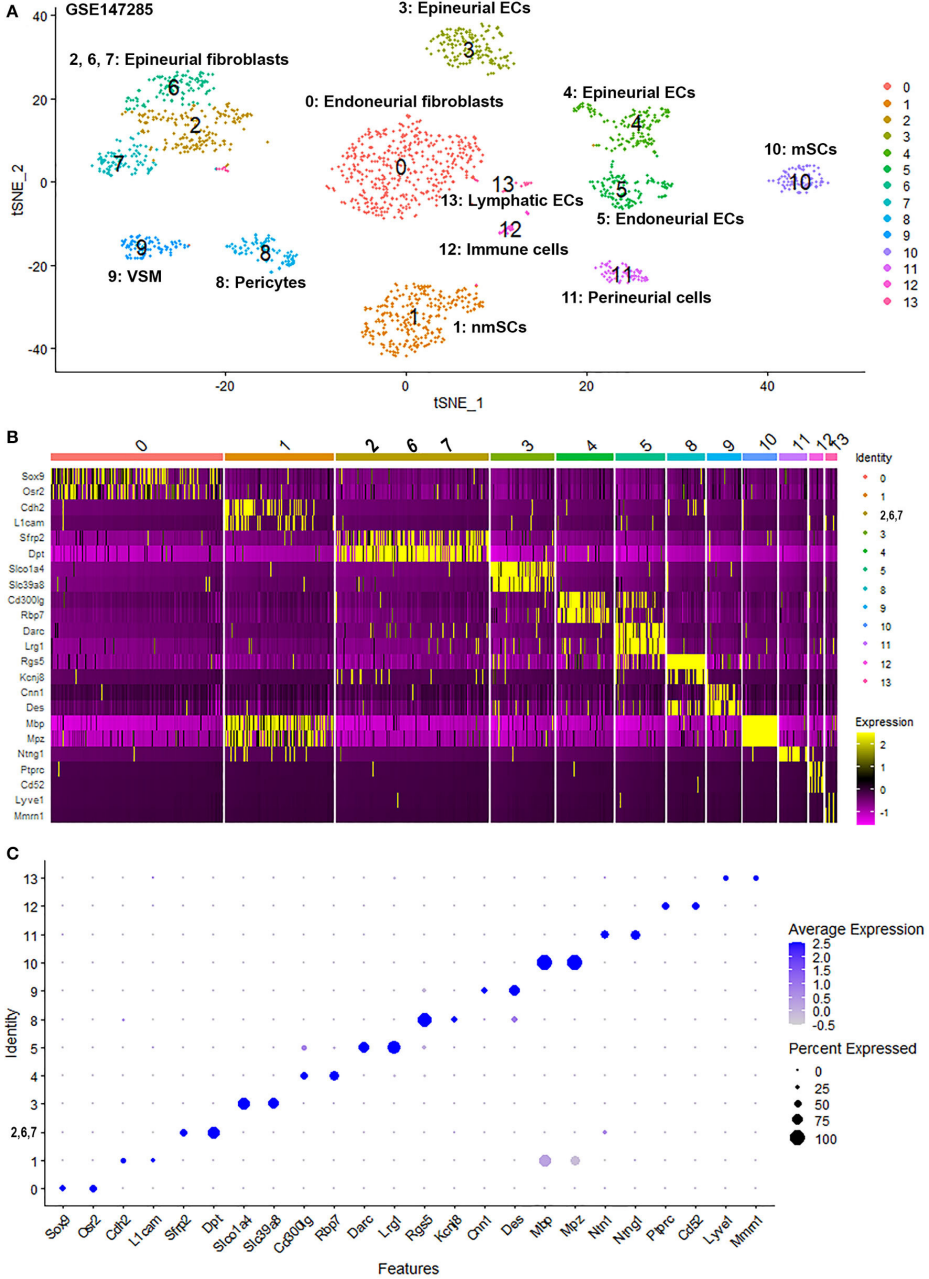
Single cell transcriptomics (GSE147285) defines cellular phenotypes in intact mouse sciatic nerve. **(A)** tSNE visualization of cell clusters in intact mouse sciatic nerve. 0: Endoneurial fibroblasts; 1: Non-myelinating Schwann cells (nmSCs); 2, 6, 7: Epineurial fibroblasts; 3, 4: Epineurial endothelial cells; 5: Endoneurial endothelial cells; 8: Pericytes; 9: Vascular smooth muscle (VSM) cells; 10: Myelinating Schwann cells (mSCs); 11: Perineurial cells; 12: Immune cells (resident macrophages, mast cells, T/NK cells); 13: Lymphatic endothelial cells. **(B)** Heat map of selected marker genes for each cell cluster, cluster 2, 6, and 7 has been merged. **(C)** Dot plot of selected marker genes for each cell cluster, cluster 2, 6, and 7 has been merged.

**Figure 2:**
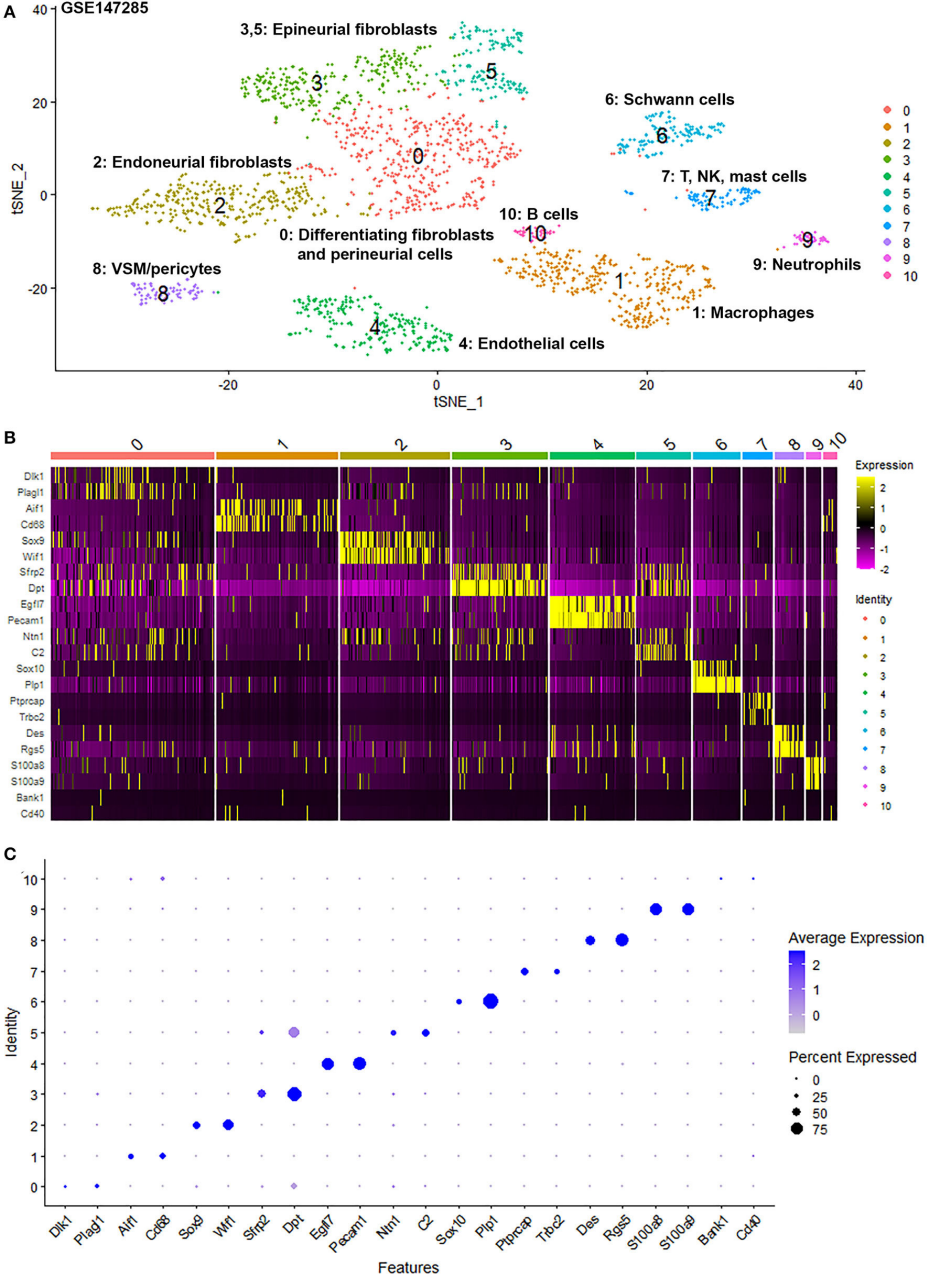
Single cell transcriptomics (GSE147285) defines cellular phenotypes in day 3 post-injury distal mouse sciatic nerve. **(A)** tSNE visualization of cell clusters in day 3 post-injury distal mouse sciatic nerve. 0: Differentiating fibroblasts and perineurial cells; 1: Macrophages; 2: Endoneurial fibroblasts; 3 and 5: Epineurial fibroblasts; 4: Endothelial cells; 6: Schwann cells; 7: T, NK and mast cells; 8: VSM/pericytes; 9: Neutrophils; 10: B cells. **(B)** Heat map of selected marker genes for each cell cluster. **(C)** Dot plot of selected marker genes for each cell cluster.

**Figure 3:**
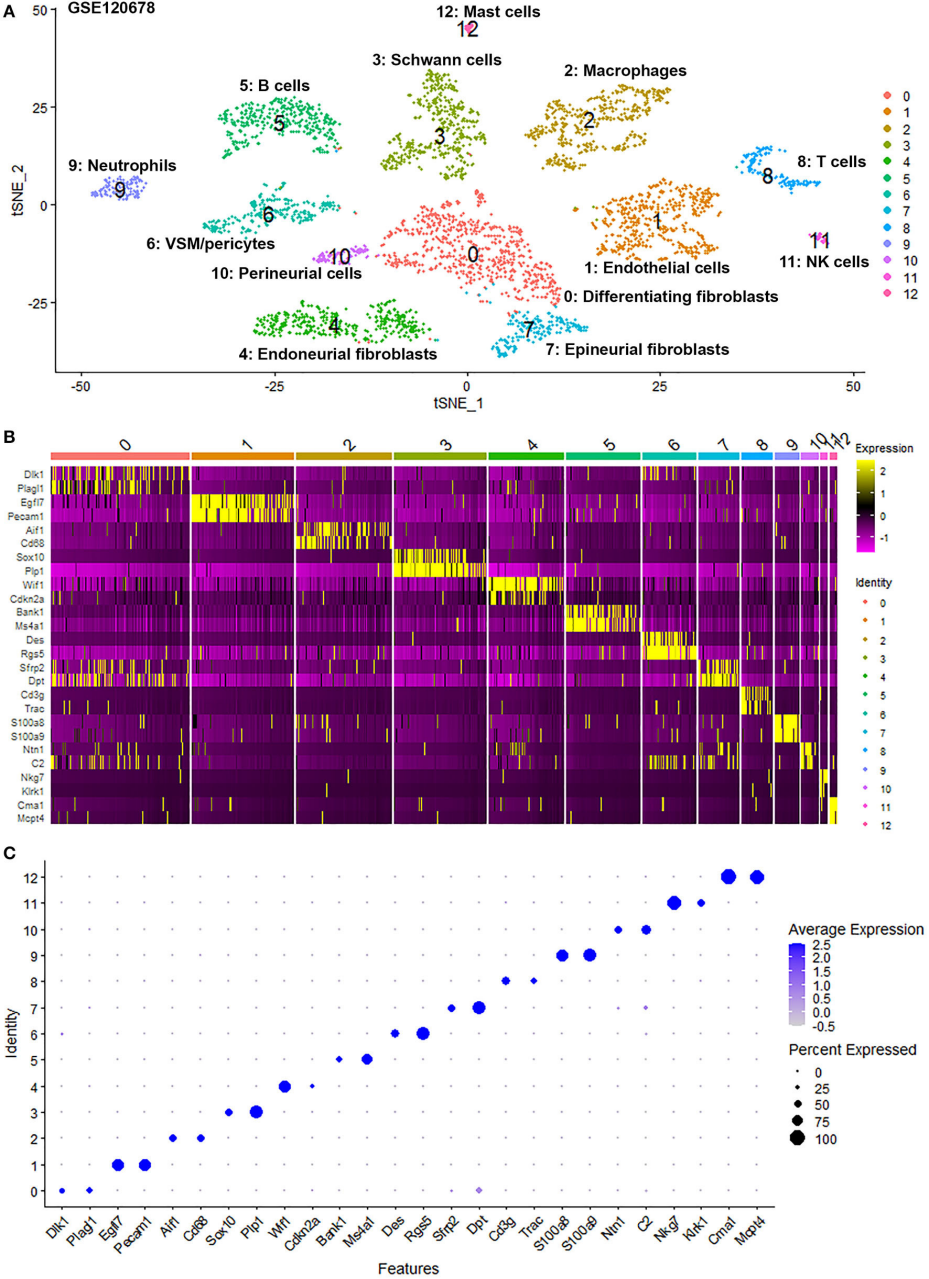
Single cell transcriptomics (GSE120678) defines cellular phenotypes in day 9 post-injury distal mouse sciatic nerve. **(A)** tSNE visualization of cell clusters in day 9 post-injury distal mouse sciatic nerve. 0: Differentiating fibroblasts; 1: Endothelial cells; 2: Macrophages; 3: Schwann cells; 4: Endoneurial fibroblasts; 5: B cells; 6: VSM/pericytes; 7: Epineurial fibroblasts; 8: T cells; 9: Neutrophils; 10: Perineurial cells; 11: NK cells; 12: Mast cells. **(B)** Heat map of selected marker genes for each cell cluster. **(C)** Dot plot of selected marker genes for each cell cluster.

To generate an unbiased cellular map of the peripheral nerve in homeostasis and regeneration at single cell resolution, we analyzed the data sets GSE147285 and GSE120678 with the R-package Seurat v3.2.1 in R v.4.0.2. Data were filtered prior to normalization and dimensional reduction by principal component analysis. This resulted in a total of 1936 cells with 14,993 features for the intact mouse sciatic nerve, a total of 2,231 cells with 16,888 features for the nerves at 3 days post-injury, and a total of 3,894 cells with 16,937 features for the nerves at 9 days post-injury. Cell transcriptomes were then normalized using SCTransform. This method is effective at normalizing scRNAseq data and allows potentially a higher number of principal components to be used for downstream analysis and clustering (Hafemeister and Satija, 2019). Principal component (PC) analysis and clustering was then undertaken using highly variable genes. Previously, Carr et al. assigned the lowest resolution at 0.4 for conservative analysis of all three datasets (Carr et al., 2019). We found that resolution 0.4 could distinguish distinct cell types in both post-injury day 3 and day 9 nerve samples (Figures 2, 3), as defined by established marker genes. However, the cell cluster of lymphatic endothelial cells, which has been reported by Wolbert et al. recently (Wolbert et al., 2020), was not labeled in intact nerves with a resolution of 0.4. We therefore increased the clustering resolution to 0.75 in order to effectively separate the cluster of lymphatic endothelial cells from other cell clusters. The increase of resolution to 0.75 also divided the epineurial fibroblast cluster into three sub-clusters (cluster 2, 6, 7 in Figure 1A). Our analysis resulted in 14 cell clusters in intact nerves (Figure 1A), 11 cell clusters in post-injury day 3 nerves (Figure 2A) and 13 cell clusters in post-injury day 9 nerves (Figure 3A). Differentially expressed genes (DEGs) for each cell cluster were identified through the analysis (Supplementary Tables 1–3).

To determine the identity of each cell cluster, we examined the expression of well-known cell type specific marker genes in cells of both intact and injured peripheral nerves (Carr et al., 2019; Toma et al., 2020; Wolbert et al., 2020). t-SNE gene expression overlays, heatmaps and dot plots were used to visualize the expression of the cell type marker genes in each cluster. In agreement with previous findings analyzing cell types in both intact and injured mouse sciatic nerve (Carr et al., 2019; Toma et al., 2020; Wolbert et al., 2020), our approach identified similar cell clusters in both intact and injured nerves (Figures 1–3). Based upon the cell function in peripheral nerve homeostasis and regeneration, we classified them as four categories of cells: Schwann cells, nerve fibroblasts, immune cells and cells associated with blood supply to the nerve. However, our analysis shows four sub-clusters of endothelial cells in the intact nerve (cluster 3, 4, 5, and 13 in Figure 1A), and in addition we identified a cluster of neutrophils in injured nerves (cluster 9 in Figures 2A, 3A), which have not been reported in previous analyses (Carr et al., 2019; Toma et al., 2020; Wolbert et al., 2020).

### Identifying Epineurial and Endoneurial Endothelial Cells in Intact Mouse Sciatic Nerve

In the intact nerve, in addition to the lymphatic EC cluster (cluster 13, Figure 1A) identified by marker genes for lymphatic ECs such as Lyve1, Mmrn1, Prox1, and Flt4 (Supplementary Figure 1) (Engelbrecht et al., 2020; Fujimoto et al., 2020; Wolbert et al., 2020), our analysis shows three distinct sub-clusters of blood vessel ECs in the intact nerve not described in the previous studies (cluster 3, 4, and 5, Figure 1A). All three new EC sub-clusters express classic EC marker genes such as Pecam1/Cd31, Tie1, and Emcn (Supplementary Figure 2) (Zhao et al., 2018; Carr et al., 2019; Kalluri et al., 2019; Toma et al., 2020; Wolbert et al., 2020). Previous studies using scRNA-seq to analyse endothelial cell profiles of specific structures of the blood supply system such as the aorta, the hematopoietic niche, the lymph nodes and the heart, often resulted in the identification of 2–4 sub-clusters of endothelial cells. These studies showed that it can be very difficult to name individual sub-clusters of EC cells because of overlapping expression patterns of key endothelial cell genes (Kenswil et al., 2018; Feng et al., 2019; Kalluri et al., 2019; Engelbrecht et al., 2020; Fujimoto et al., 2020).

**Figure 4:**
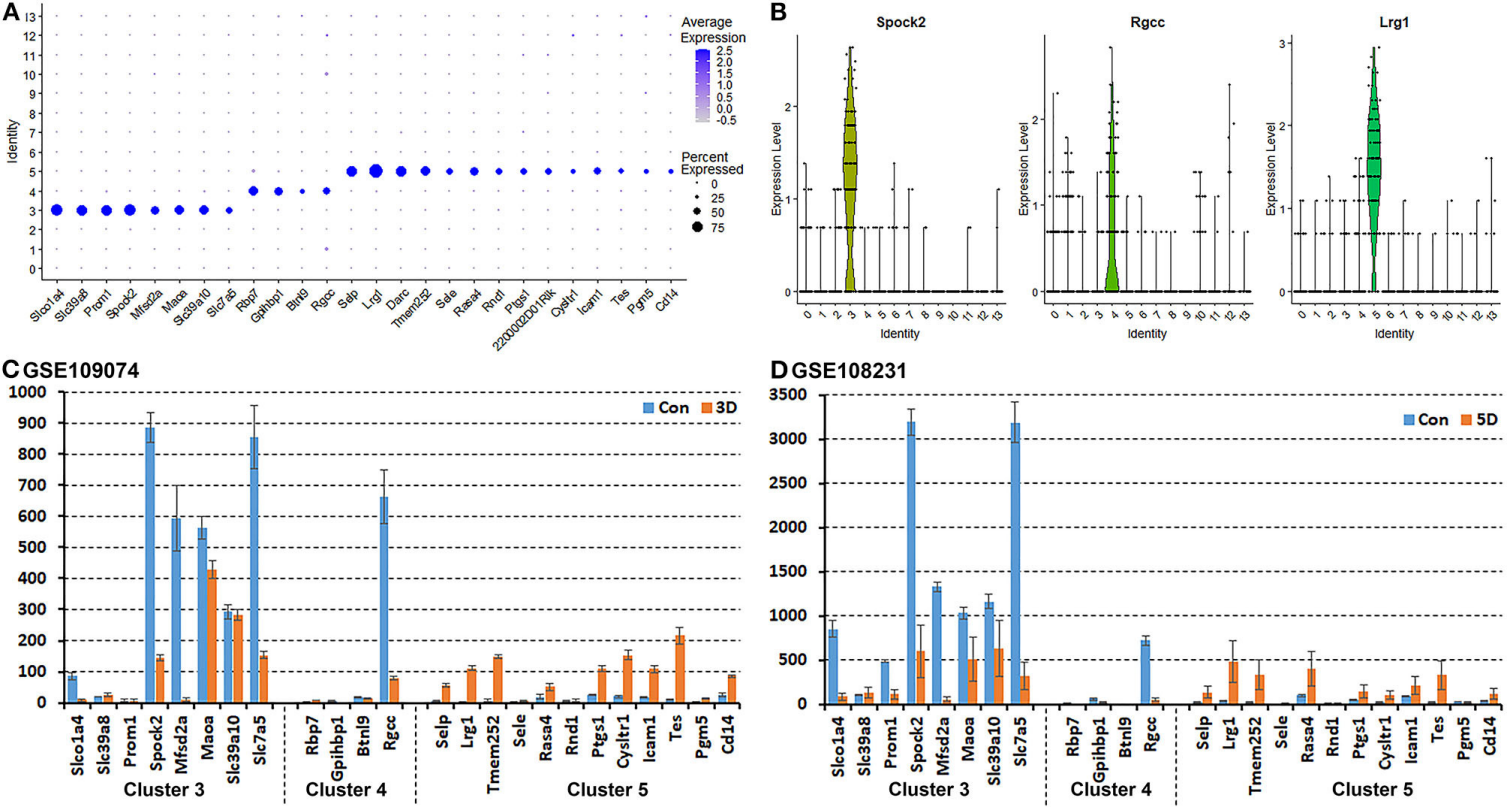
Identification of DEGs unique to cluster 3, 4, and 5 in the intact mouse sciatic nerve. **(A)** Dot plot showing unique DEGs for cluster 3, 4, and 5 in the intact sciatic nerve. **(B)** Violin plot showing Spock2 expression in cluster 3, Rgcc in cluster 4 and Lrg1 in cluster 5 ECs in the intact sciatic nerve. **(C)** mRNA count showing the expression level of unique DEGs for cluster 3, 4, and 5 in intact sciatic nerve and in the distal nerve at day 3 post crush injury. **(D)** mRNA count showing the expression level of unique DEGs for cluster 3, 4, and 5 in intact sciatic nerve and in the distal nerve at day 5 post crush injury.

It has been proposed that ECs of the blood vessels show remarkable heterogeneity and their phenotypes vary in time and space, differ in structure and function, and change in health and disease (Aird, 2012); this might explain why the EC sub-types can be difficult to identify in scRNA-seq studies. To name the subtype of ECs in cluster 3, 4 and 5, we searched DEGs (Supplementary Table 1) that was identified during cell clustering analysis in order to find unique DEGs for cluster 3, 4, and 5 respectively. This analysis revealed that Slco1a4, Slc39a8, Prom1, Spock2, Mfsd2a, Maoa, Slc39a10, and Slc7a5 are unique DEGs for cluster 3 (p ≤ 3.56008242907399E-123). Rbp7, Gpihbp1, Btnl9, and Rgcc are unique DEGs for cluster 4 (p ≤ 5.82149130420303E-45). Selp, Lrg1, Darc, Tmem252, Sele, Rasa4, Rnd1, Ptgs1, 2200002D01Rik, Cysltr1, Icam1, Tes, Pgm5, and Cd14 are unique DEGs for cluster 5 (*p* ≤ 4.27728189243353E-59) (Figure 4A). Next, we used bulk mRNA sequencing data sets GSE109074 (Gokbuget et al., 2018) and GSE108231 (Norrmen et al., 2018) to determine the expression level of above DEGs in the intact mouse sciatic nerve in order to select appropriate marker genes for *in vivo* validation by immunostaining. Bulk mRNA sequencing data sets analysis showed that Spock2, Slc7a5, Mfsd2a, Maoa, Slc39a10, Slco1a4, and Rgcc are highly expressed in the intact mouse sciatic nerve (Figures 4C,D). We therefore chose Spock2 for cluster 3 and Rgcc for cluster 4 to validate their *in vivo* expression in ECs by immunostaining (Figure 4B). All the unique DEGs for cluster 5 are weakly expressed in the intact mouse sciatic nerve (Figures 4C,D). Lrg1 has been shown to promote new blood vessel growth by modulating Tgfβ signaling in endothelial cells (Wang et al., 2013). We chose Lrg1 for cluster 5 to do *in vivo* staining based on the availability of a good Lrg1 antibody (Figure 4B). Interestingly, most unique DEGs for cluster 3 and cluster 4 were down-regulated following injury while most unique DEGs for cluster 5 were up-regulated in response to injury (Figures 4C,D). This might explain why all ECs have been placed into just one cluster in both day 3 post injury (cluster 4 in Figure 2A) and day 9 post injury (cluster 1 in Figure 3A) because they lose their sub-cluster identity following injury.

**Figure 5:**
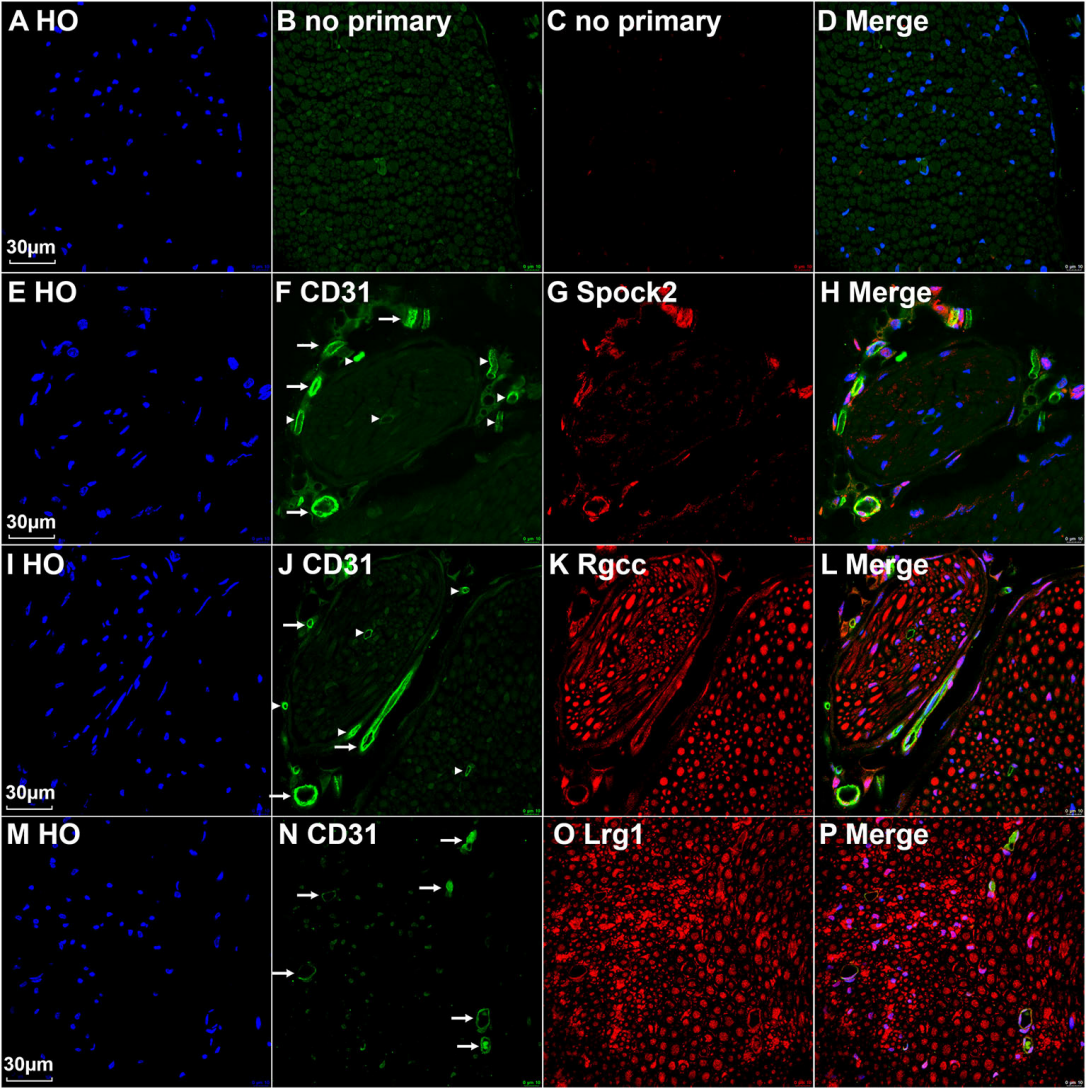
Identify epineurial and endoneurial endothelial cells in intact mouse sciatic nerve by immunostaining. **(A–D)** Control slides staining without adding primary antibodies in the blocking buffer. **(E–H)** Double staining CD31 with Spock2 showing that Spock2 is strongly expressed in ECs of large diameter blood vessels outside the epineurium (indicated by arrows). Arrowheads show no expression. **(I–L)** Double staining CD31 with Rgcc showing that Rgcc is strongly expressed in ECs of large diameter blood vessels outside the epineurium (indicated by arrows). Arrowheads show no expression. Rgcc is also strongly expressed in axons inside the endoneurium. **(M–P)** Double staining CD31 with Lrg1 showing that Lrg1 is expressed in ECs of endoneurium (indicated by arrows). Lrg1 is also strongly expressed in axons inside the endoneurium. Scale bars in **(A)**, **(E)**, **(I)**, and **(M)** 30 μm.

All cells in cluster 3, 4, and 5 express the classic EC marker gene Pecam1/Cd31 (Supplementary Figure 2). We then double stained CD31 with Spock2, Rgcc or Lrg1 to reveal the difference of their expression pattern in intact mouse sciatic nerve. Control sections without adding primary antibodies only showed weak background staining (Figures 5A–D). Staining Spock2 and Rgcc showed that both Spock2 and Rgcc are strongly expressed in ECs of large diameter blood vessels outside the epineurium (Figures 5E–L, indicated by arrows). The expression of both Spock2 and Rgcc are hardly detectable in ECs of endoneurium (Figures 5E–L, indicated by arrowheads). In contrast, Lrg1 is expressed in ECs of endoneurium (Figures 5M–P, indicated by arrows). In the intact sciatic nerve, Spock2 is only expressed in ECs outside the epineurium (Figures 5E–H), however, both Rgcc and Lrg1 also show strong expression as round dots inside the endoneurium which is, the typical morphology of peripheral axons (Figures 5I–P). According to the expression pattern of Spock2, Rgcc, and Lrg1 in the intact mouse sciatic nerve, we named cluster 3 and cluster 4 as epineurial ECs, and named cluster 5 as endoneurial ECs (Figure 1A).

**Figure 6:**
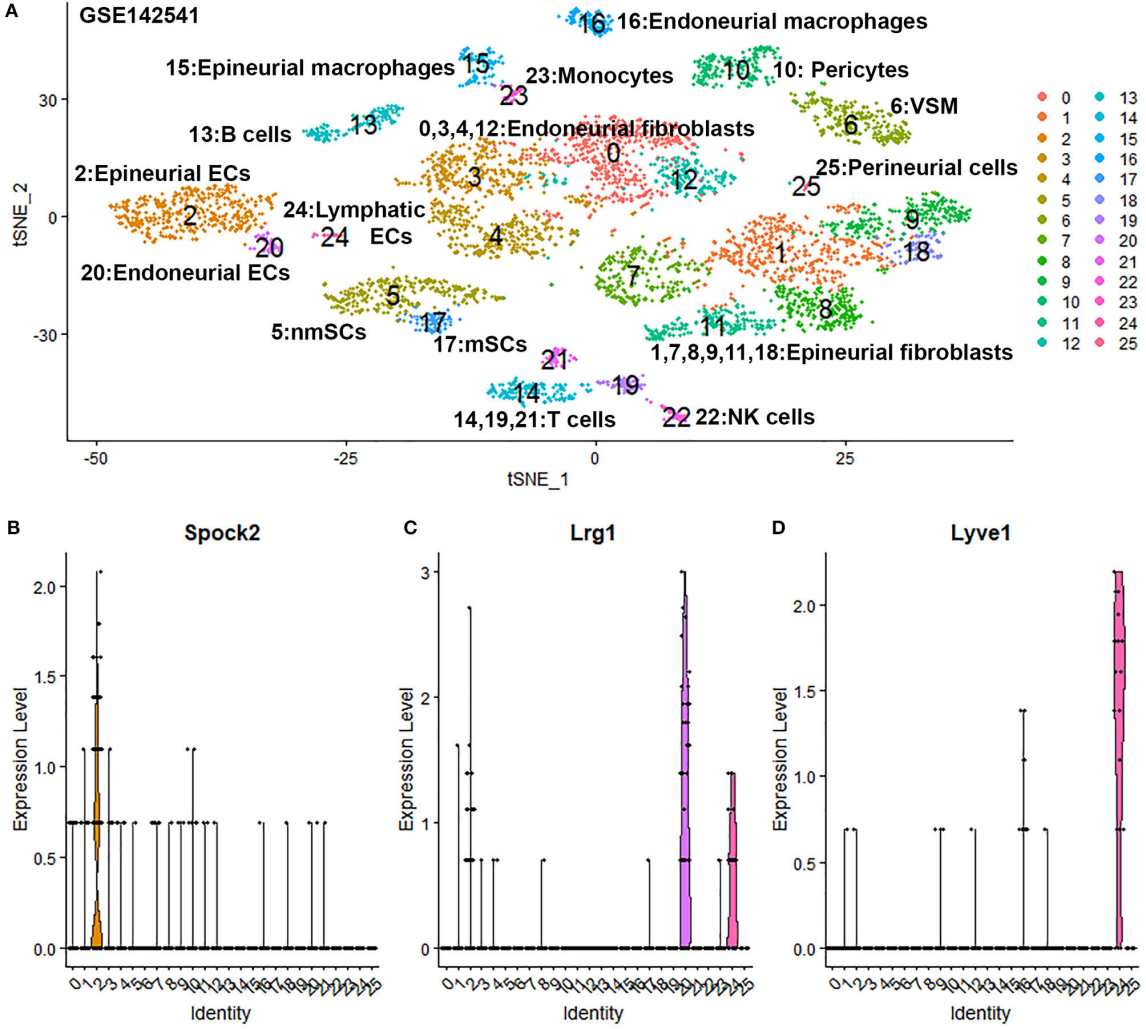
Single cell transcriptomics (GSE142541) defines cellular phenotypes in intact mouse sciatic nerve and the brachial nerve plexus. **(A)** tSNE visualization of 26 cell clusters in intact mouse sciatic nerve at resolution 1.35. **(B)** Violin plot showing Spock2 expression in the epineurial EC cluster (cluster 2). **(C)** Violin plot showing Lrg1 expression in the endoneurial EC cluster (cluster 20). **(D)** Violin plot showing Lyve1 expression in the lymphatic EC cluster (cluster 24).

To confirm our finding that ECs in the intact mouse peripheral nerves could be grouped into epineurial, endoneurial and lymphatic sub-types, we further analyzed another scRNA-seq data set GSE142541 generated to analyse cell types in the intact mouse sciatic nerve and the brachial nerve plexus (Wolbert et al., 2020). This data set contains more cells but lower number of genes per cell compared to data set GSE147285. Cells were filtered using the following conditions: number of features per cell 200–2,000 and percent mitochondrial DNA content per cell <8%. This resulted in a total of 5,188 samples with 15,057 features. In contrast to the data set GSE147285, our analysis for GSE142541 only show three EC sub-clusters (cluster 2, 20 and 24 in Figure 6A), possibly due to the lower number of genes per cell. Using the above marker genes, we identify cluster 2 as epineurial ECs, cluster 20 as endoneurial ECs, and cluster 24 as lymphatic ECs (Figure 6A). Examining DEGs for both cluster 3 and 4 of data set GSE147285 showed that they are also expressed epineurial EC cluster genes found in the GSE142541 data set (cluster 2) (Supplementary Table 4) such as Sox17 and Spock2 (Figure 6B). This data set analysis further confirmed our finding from immunostaining that cluster 3 and 4 in data set GSE147285 are epineurial ECs (Figure 1A). Lrg1 remains a DEG for endoneurial ECs (Figure 6C), and Lyve1 remains a DEG for lymphatic ECs (Figure 6D). The GSE142541 data set contains cells from the brachial nerve plexus in addition to cells from sciatic nerves (Wolbert et al., 2020). Thus, analyzing this data set showed that ECs in the mouse peripheral nerves could be grouped into lymphatic, endoneurial and epineurial sub-types by scRNA data analysis.

### Identifying Endoneurial Fibroblasts and Perineurial Cells in Data Set GSE142541

**Figure 7:**
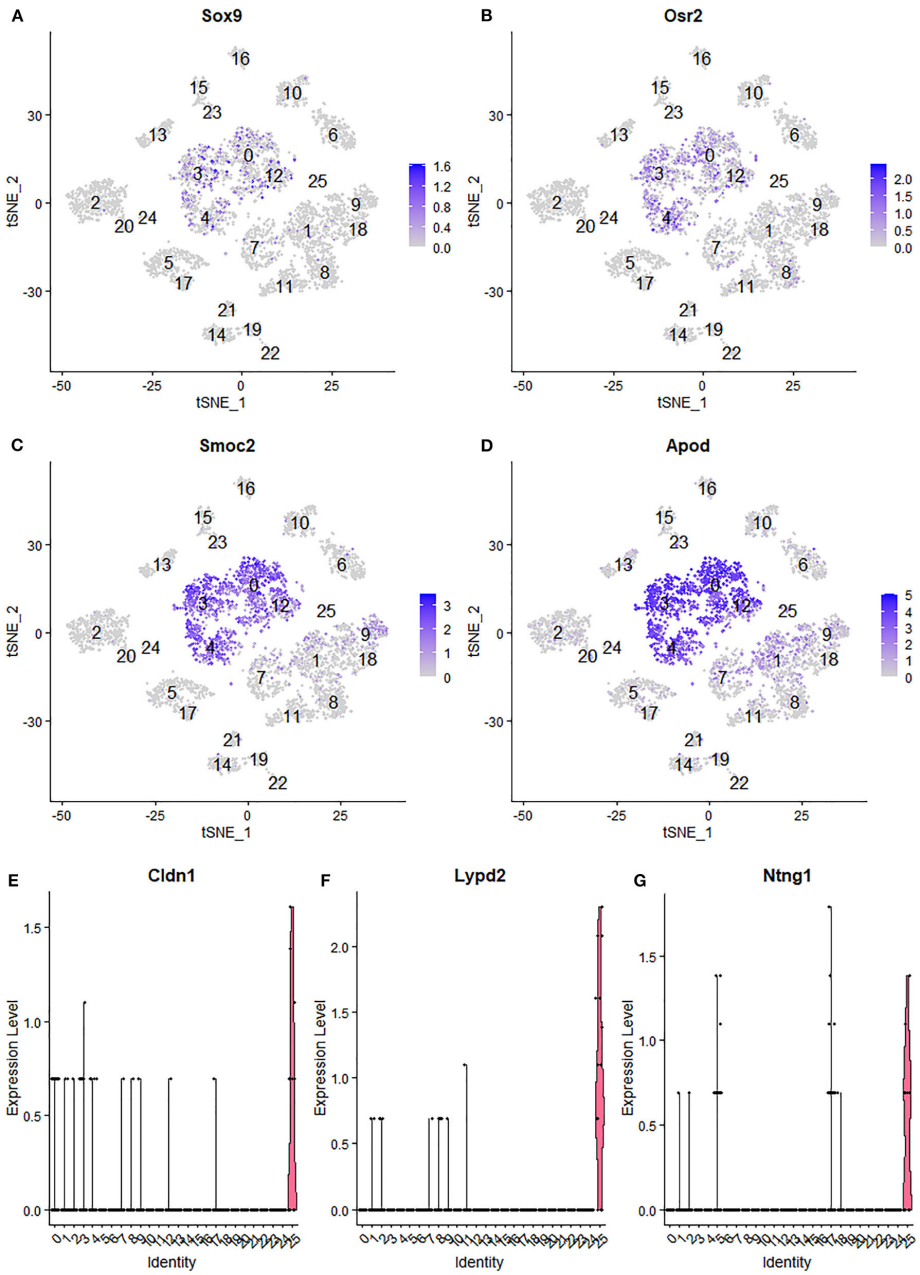
Identifying endoneurial fibroblasts and perineurial cells in data set GSE142541. **(A,B)** tSNE plot showing Sox9 and Osr2 expression in endoneurial fibroblasts (cluster 0, 3, 4, and 12). **(C,D)** tSNE plot showing Smoc2 and Apod expression in endoneurial fibroblasts. **(E,F)** Violinplot showing Cldn1, Lypd2, and Ntng1 expression in perineurial cells (cluster 25).

Due to endoneurial fibroblasts expressing Ngfr (Stierli et al., 2018), in the paper by Wolbert et al., 2020, they named a cluster of endoneurial fibroblasts as nmSCs in their analysis, therefore suggested Smoc2 and Apod, which are also expressed in this mis-labeled cluster, as marker genes for nmSCs (Wolbert et al., 2020). By re-analysis the data set GSE142541, we identified cluster 0, 3, 4 and 12 as endoneurial fibroblasts (Figure 6A) as they express marker genes for endoneurial fibroblasts such as Sox9, Osr2, Pdgfra, Cd34, Abca9, Cdkn2a, Cdkn2b, and Plxdc1 (Figures 7A,B, Supplementary Table 4). They also express high levels of Smoc2 and Apod (Figures 7C,D). Instead, we identify myelinating Schwann cells (mSC) as cluster 17 and nmSCs as cluster 5 (Figure 6A). Cluster 25 express high levels of Cldn1, Lypd2, Ntng1, Slc2a1, and Itgb4 (Figures 7E–G), and we identified cluster 25 as perineurial cells (Figure 6A), which previously have been named as a novel cluster of endothelial cells (Wolbert et al., 2020). Because of the high number of cells for this data set, immune cells could be clustered into B cells (cluster 13 in Figure 6A), epineurial macrophages (cluster 15 in Figure 6A), endoneurial macrophages (cluster 16 in Figure 6A), T cells (cluster 14, 19 and 21 in Figure 6A) and NK cells (cluster 22 in Figure 6A).

### Identifying the Cluster of Neutrophils in Injured Nerves

**Figure 8:**
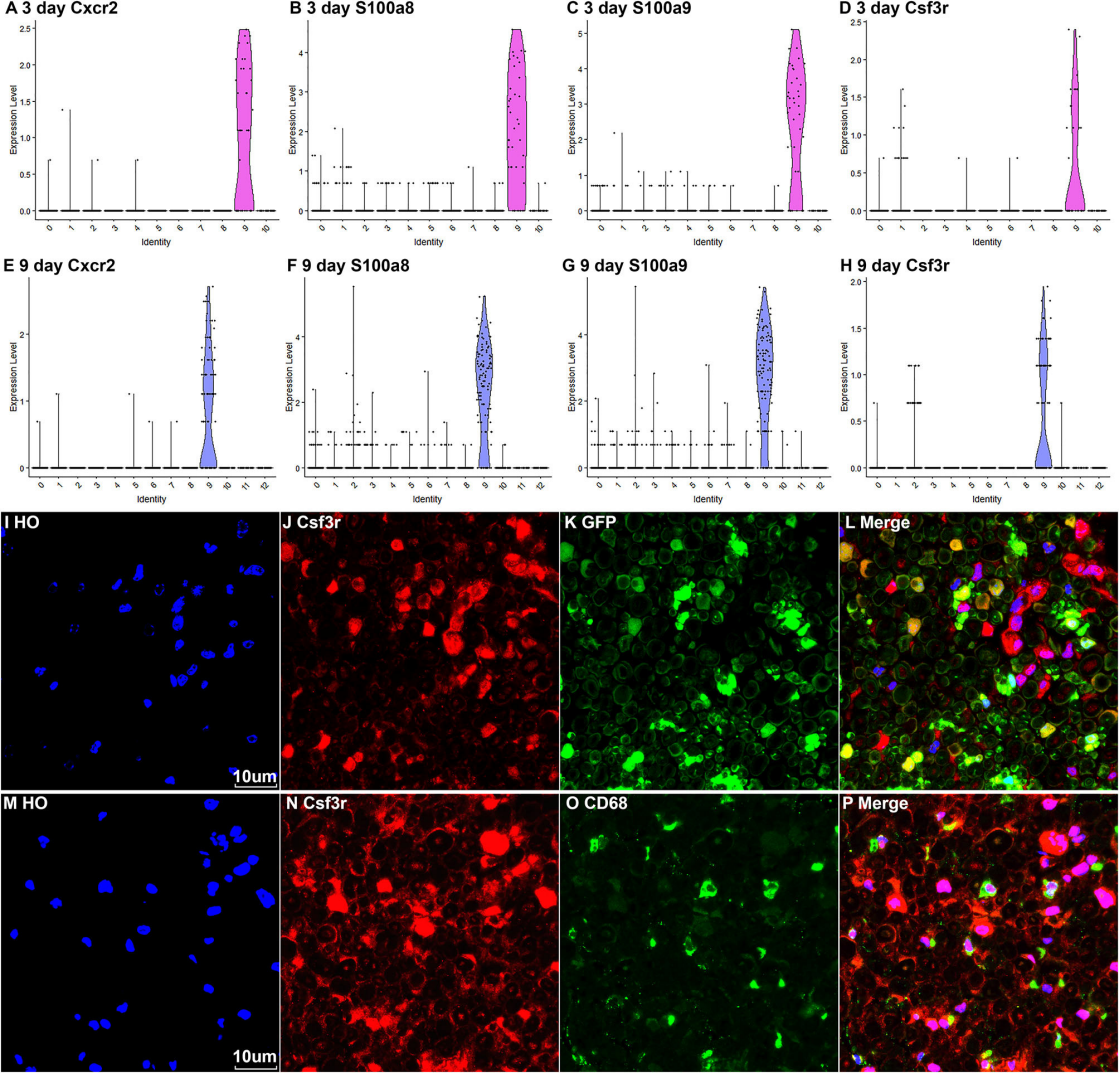
Marker genes for identifying the cluster of neutrophils in injured nerves. **(A–D)** Violin plot of selected marker genes Cxcr2, S100a8, S100a9, and Csf3r for neutrophils at day 3 post-injury. **(E–H)** Violin plot of selected marker genes Cxcr2, S100a8, S100a9, and Csf3r for neutrophils at day 9 post-injury. **(I–L)** Csf3r staining showing neutrophils in the distal nerve of PLP-GFP mice at day 3 post injury. **(M–P)** Csf3r and Cd68 double staining showing neutrophils in the distal nerve of C57BL/6 mice at day 3 post injury. Scales bars in **(I)** and **(M)** 10 μm.

Neutrophils are the early inflammatory cells infiltrating the injury site and the distal nerve for debris clearance (Lindborg et al., 2017). Previous studies have shown that the number of neutrophils increases rapidly after injury and can be detected at just 8 h after injury with a peak at 24 h post-injury (Perkins and Tracey, 2000; Barrette et al., 2008; Lindborg et al., 2017). However, the cluster of neutrophils was not identified in Carr et al. and Toma et al.'s reports because their studies focused on the cell population of fibroblasts (Carr et al., 2019; Toma et al., 2020). Recently scRNA-seq data analysis in other tissues showed that S100a8, S100a9, Cxcr2, and Cxcl2 are effective marker genes to identify neutrophils (Evrard et al., 2018; Xie et al., 2020). We tested these marker genes in the injured mouse sciatic nerve and identified cluster 9 in both post-injury day 3 and post-injury day 9 nerves as neutrophils (Figures 2A, 3A, 8A–H). Examining the DEGs for nerves at day 3 post injury (Supplementary Table 2) revealed that top 10 suggested marker genes for cluster 9 are Cxcr2, Trem1, S100a9, Il1b, S100a8, Trem3, Clec4e, Nlrp3, Il1r2, and Csf3r (all p vablues ≤ 1.06160807720519E-79).

The Csf3r gene encodes the colony-stimulating factor 3 receptor that is critical for differentiation and proliferation of neutrophils (Maxson and Tyner, 2017). Mutation of Csf3r activates the receptor and promotes neutrophil proliferation, leading to chronic neutrophilic leukemia (Duployez et al., 2019). We then select Csf3r as a marker to validate its expression on neutrophils using immunostaining on mouse distal sciatic nerve sections at day 3 post injury. Staining for Csf3r on distal nerve sections from PLP-GFP mice at day 3 post injury showed that Csf3r is expressed by large cells localized between GFP positive Schwann cells (Figures 8I–L). Double staing Csf3r with a marophage marker CD68 indiated that Csf3r is expressed on a diffrenct cell population from macrophages in the distal nerve (Figures 8M–P). In particularly, Csf3r positive cells exhibit segmented nuclei with three to five distinct lobes connected by thin filaments (Figures 8I,M), this is a key feature of mature neutrophils (Hermann and Gunzer, 2019). Thus, the Csf3r immunostating confirms that Csf3r is a reliable marker gene to identify neutrophils in the distal nerve stump.

### Proliferating Cells in the Distal Nerve Stump Following Injury

**Figure 9:**
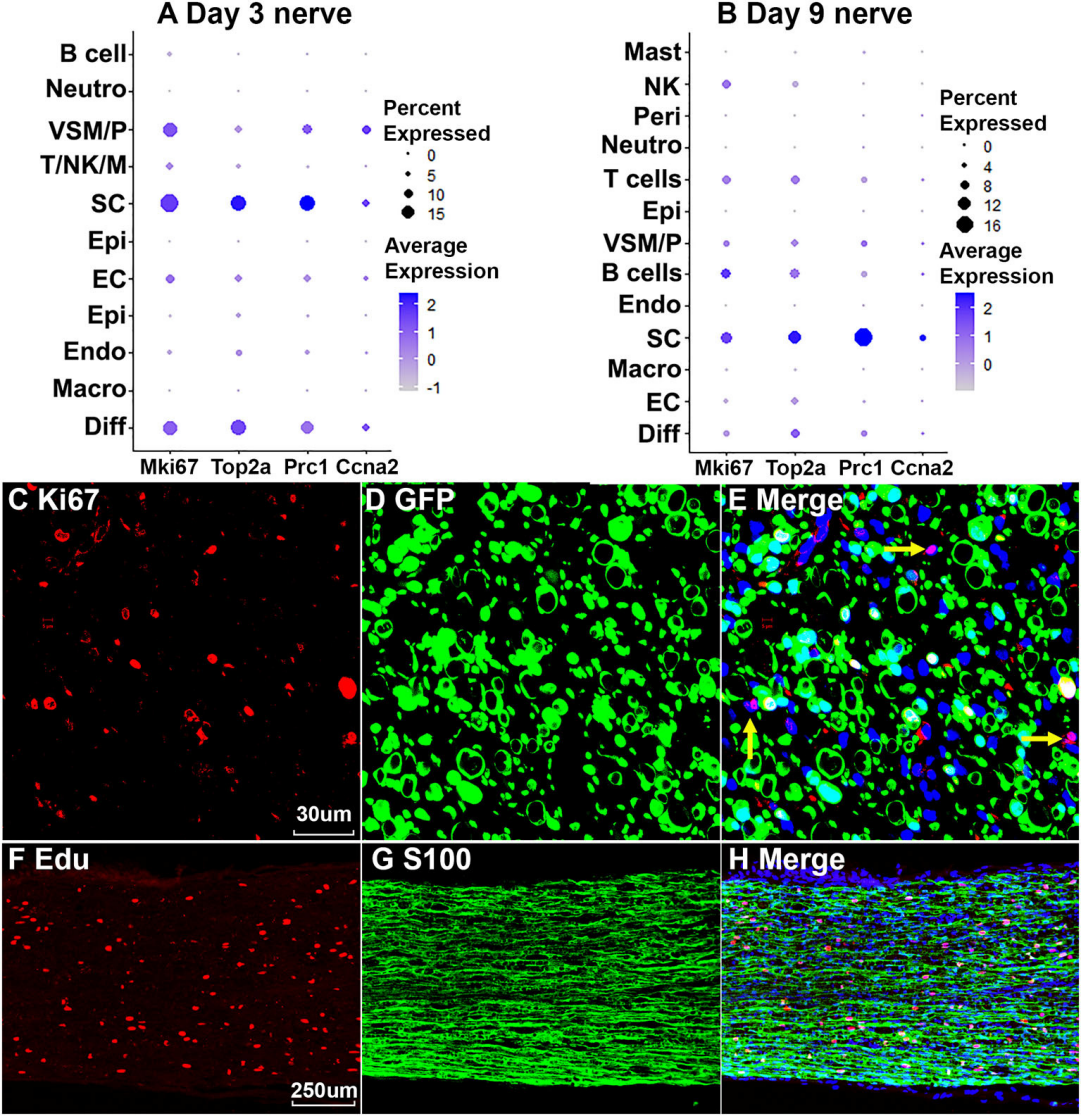
Proliferating cells in the injured peripheral nerves. **(A)** Marker genes Mki67, Top2a, Prc1, and Ccna2 identify Schwann cells as the most proliferative cells in the distal nerve at day 3 post injury. **(B)** Mki67, Top2a, Prc1, and Ccna2 identify Schwann cells as the most proliferative cells in the distal nerve at day 9 post injury. **(C–E)** Mki67/Ki67 staining showing Schwann cells (GFP positive) are the most proliferative cells in the distal nerve at day 7 post injury in PLP-GFP mice. Yellow arrows in **(E)** show proliferation of non-Schwann cells (GFP negative). **(F–H)** EdU labeling showing that Schwann cells (S100 positive) are the most proliferative cells in the distal nerve at day 7 post injury in C57 mice. Scales bar in **(C)** 30, in **(F)** 250 μm.

Cells proliferate in the distal nerve stump following peripheral nerve injury, we then identify proliferating cells in the distal nerve stump using well characterize marker genes Mki67/Ki67, Top2a, Prc1, and Ccna2 for proliferating cells (Stierli et al., 2018; Carr et al., 2019; Dun et al., 2019; Toma et al., 2020; Wolbert et al., 2020). At day 3 post injury, Mki67/Ki67, Top2a, Prc1, and Ccna2 marker genes identify Schwann cells as the most proliferative cells in the distal nerve stump (Figure 9A). Fibroblasts, vascular smooth muscle (VSM) cells and pericytes are also highly proliferative in the distal nerve stump at day 3 post injury (Figure 8A). ECs proliferate as well at day 3 post injury but with much lower percentage of cells proliferating (Figure 9A). At day 3 post injury, 19.85% Schwann cells, 15.76% fibroblasts, 15.29% VSM and pericytes, and 7.87% ECs express Mki67/Ki67 (Figure 9A). At day 9 post injury, Schwann cells remain the most proliferative cells in the distal nerve stump (Figure 9B). In contrast to day 3 post injury, infiltrated B cells, T cells and NK cells at day 9 post injury are more proliferative than other cell types except Schwann cells (Figure 9B). At day 9 post injury, 11.34% Schwann cells, 9.42% B cells, 7.93% T cells and 7.89% NK cells express Mki67/Ki67 (Figure 9A). Having identified Schwann cells as the most proliferative cells in the distal nerve stump, we performed Ki67 staining in PLP-GFP mice as well as EdU and S100b double labeling in C57BL/6J mice to reveal proliferating cells in the distal nerve stump at day 7 post injury. Both Ki67 staining (Figures 9C–E) and EdU labeling (Figures 9F–H) results confirm that Schwann cells are the most proliferative cells in the injured mouse sciatic nerve.

### Changes in the Molecular Profile of Each Cell Type Following Peripheral Nerve Injury

Gene expression changes following peripheral nerve injury have been extensively studied by microarray analysis and bulk mRNA sequencing (Arthur-Farraj et al., 2012, 2017; Fontana et al., 2012; Jessen and Mirsky, 2016; Clements et al., 2017; Gokbuget et al., 2018; Norrmen et al., 2018; Stratton et al., 2018; Tomlinson et al., 2018; Boissonnas et al., 2020; Ydens et al., 2020). However, cell type specific gene expression changes have not been studied. We therefore performed integrated analysis for intact sciatic nerve and sciatic nerves at day 3 and day 9 post injury, followed by a detailed analysis of cell type specific differential gene expression between intact and injured nerves. Only data set GSE147285 for the intact nerves was used for this analysis because our analysis showed that GSE142541 data set contains much lower numbers of genes per cell compared to data sets GSE147285 and data set GSE120678.

**Figure 10:**
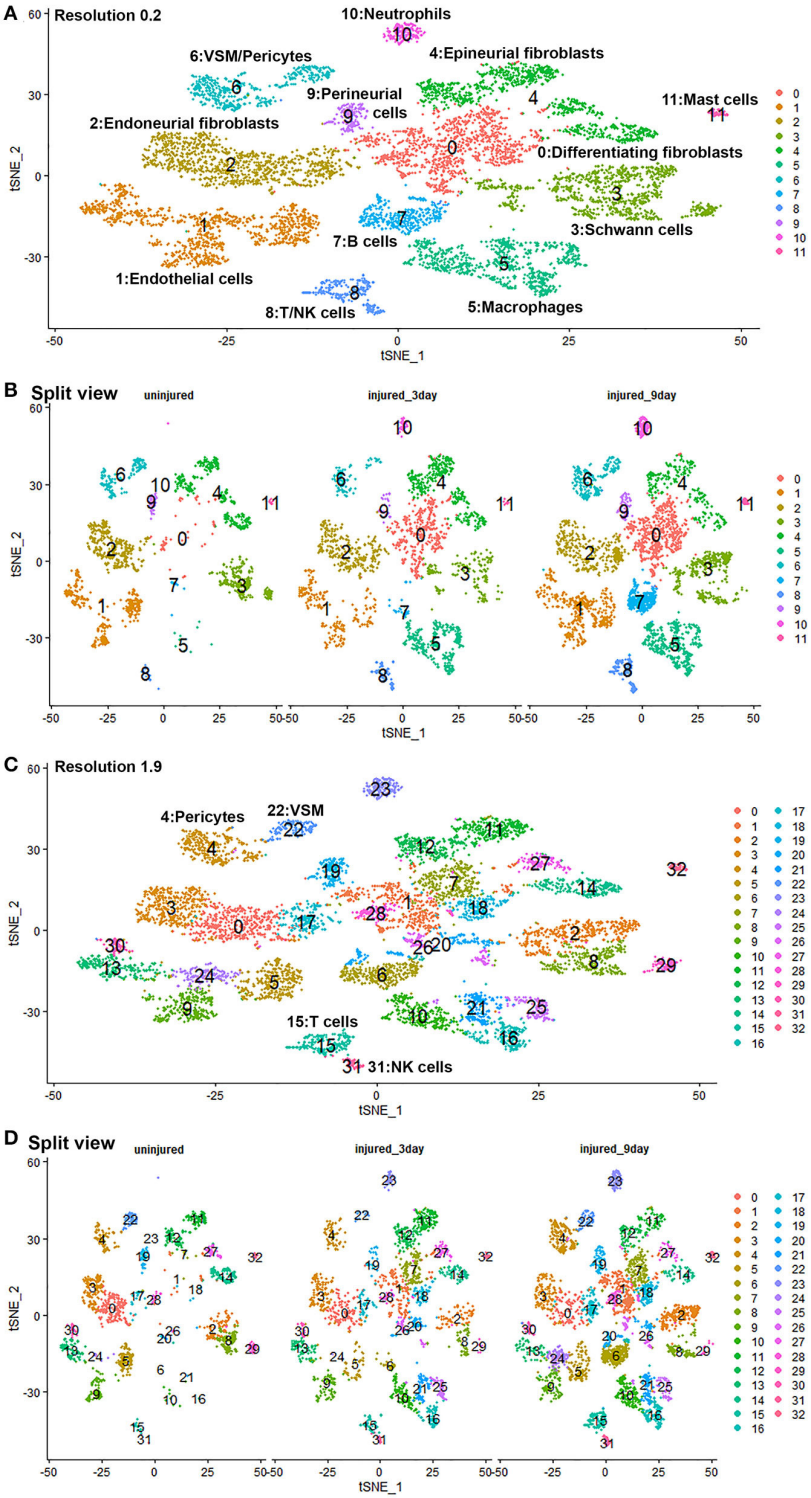
GSE147285 and GSE120678 data sets integrated analysis for intact sciatic nerve and sciatic nerves at day 3 and day 9 post injury. **(A)** tSNE visualization of cell clusters for integrated analysis at resolution 0.2. NK cells were not separated from T cells, and pericytes were not separated from VSM at resolution 0.2. **(B)** Split view of cell clusters at resolution 0.2 for intact sciatic nerve and sciatic nerves at day 3 and day 9 post injury. **(C)** tSNE visualization of cell clusters for integrated analysis at resolution 1.9. NK cells (cluster 31) were separated from T cells (cluster 15), and pericytes (cluster 4) were separated from VSM (cluster 22). **(D)** Split view of cell clusters at resolution 1.9 for intact sciatic nerve and sciatic nerves at day 3 and day 9 post-injury.

Quality control filtering was the same as described for the individual data sets. After integration of the three data sets there were 8061 cells with 37,607 genes. The three datasets are well-integrated according to the t-SNE plots while some differences between the cell populations in each condition are apparent (Figure 10). To compare gene expression changes for the main cell types, a lower clustering resolution (0.2) was used to avoid sub-clusters forming in each cell type. Using a low clustering resolution, NK cells were not separated from T cells, and pericytes were not separated from VSM, but the other cell types clustered appropriately (Figures 10A,B). We then increased the clustering resolution (1.9) in order to separate the NK cells from T cells (Figures 10C,D) and pericytes from VSM cells (Figures 10C,D). Subsequently, we compared differential gene expression between intact and injured nerves for T cells, NK cells, pericytes and VSM at resolution 1.9, and compared cell type specific differential gene expression between intact and injured nerves for other cell types at resolution 0.2. This analysis identified genes significantly up-regulated and down-regulation in Schwann cells, fibroblasts, endothelial cells, B cells, macrophages VSM and pericytes (Table 1, Supplementary Table 5).

**Table 1:** Cell type specific gene up- and down-regulation.

**Cell types**	**Intact vs 3D injury**		**Intact vs 9D injury**		**3D injury vs 9D injury**		**Total changes**
	**Up**	**Down**	**Up**	**Down**	**Up**	**Down**	
Differentiating fibroblasts	300	15	120	34	75	480	1,024
Endothelial cells	176	93	121	269	28	187	874
Endoneurial fibroblasts	1,533	153	719	191	72	521	3,189
Schwann cells	2,045	117	66	658	1,529	172	4,587
Epineurial fibroblasts	100	69	21	53	11	76	330
Macrophages	27	0	25	0	30	77	159
B cells	1	1	17	215	12	192	438
Perineurial cells	14	2	4	4	1	3	28
Pericytes	109	19	0	0	0	0	128
VSM	11	0	0	0	0	0	11

Schwann cells have the highest number of significant DEG and endoneurial fibroblasts have second highest number, supporting the view that Schwann cells and endoneurial fibroblasts are the two most important cell types promoting peripheral nerve regeneration. Our analysis showed that there were no significant gene up- or down regulation in mast cells, neutrophils, T cells and NK cells. One possible reason is the low number or lack of these immune cells in the intact nerve would impede this analysis. Another possibility is that these immune cells are recruited to the distal nerve stump to execute their function and the distal nerve environment does not significantly regulate their gene expression.

### Cell-Cell Communication in Intact and Injured Nerves

Another great advantage of analyzing scRNA-seq data is that cell-cell communication can be revealed in order to further understand physiological processes. Bioinformatic tools for cell-cell communication analysis have been developed such as CellPhoneDB (Efremova et al., 2020) and Celltalker (Cillo et al., 2020). Previously, Toma et al. analyzed cells of the distal nerve stump communicating with regenerating neurons during peripheral nerve regeneration (Toma et al., 2020). Here we report our analysis of cell-cell communication in the nerve trunk using CellPhoneDB with the cell clusters identified in intact nerves and cell clusters identified in the distal nerve at day 3 and day 9 post-injury.

**Figure 11:**
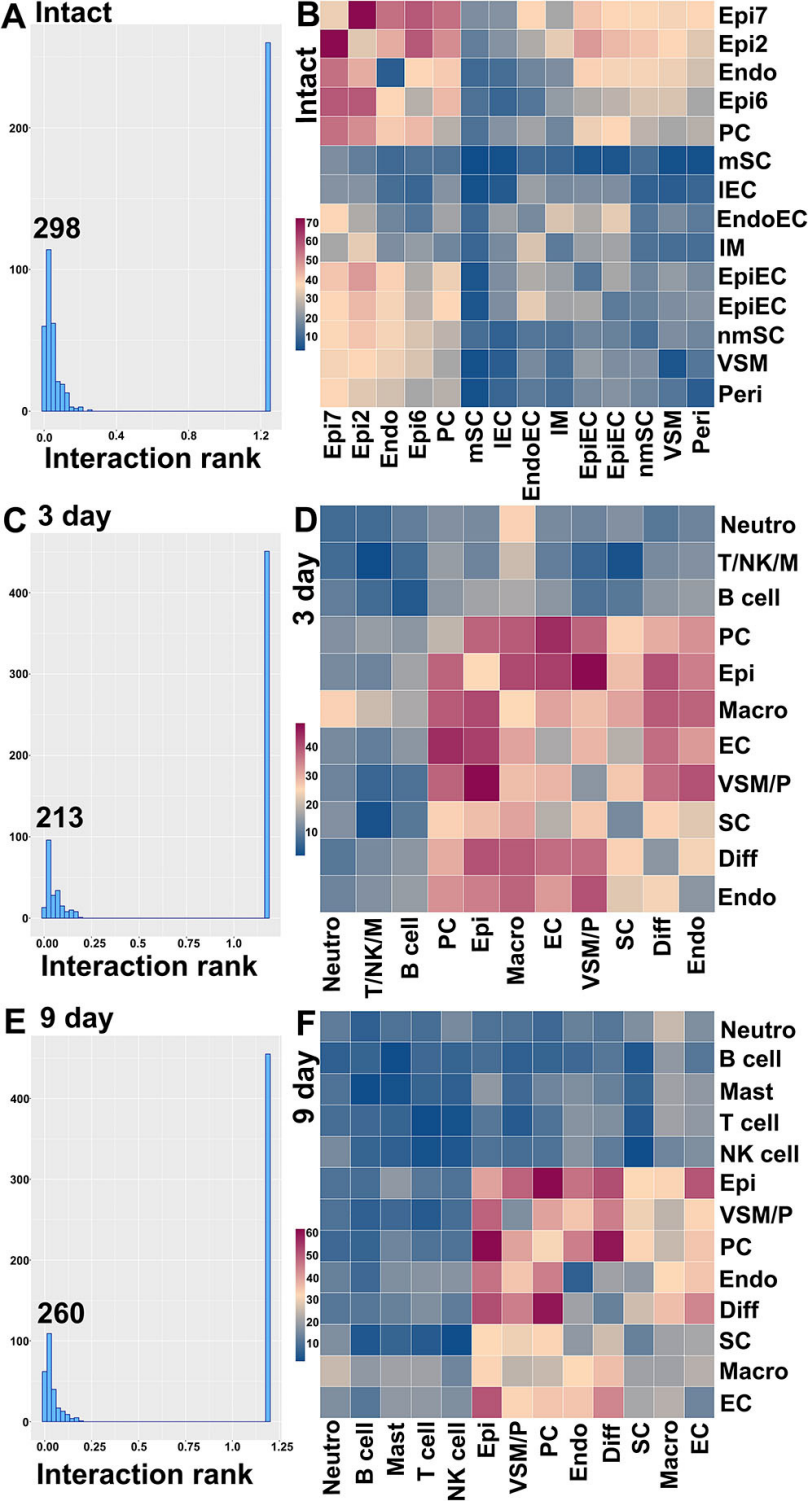
Ligand-receptor interactions between the cell clusters in intact and injuried nerves. **(A)** Distribution of CellphoneDB interaction rank for intact nerves. 298 significant ligand-receptor interactions were identified. **(B)** Heatmap of number of ligand-receptor interactions between the cell clusters in intact nerves. **(C)** Distribution of CellphoneDB interaction rank for the distal nerve at day 3 post-injury. Two hundred and thirteen significant ligand-receptor interactions were identified. **(D)** Heatmap of number of ligand-receptor interactions between the cell cluters in the distal nerve at day 3 post-injury. **(E)** Distribution of CellphoneDB interaction rank for the distal nerve at day 9 post-injury. Two hundred and sixty significant ligand-receptor interactions were identified. **(F)** Heatmap of number of ligand-receptor interactions between the cell clusters in the distal nerve at day 9 post-injury. Endo, Endoneurial fibroblasts; PC, Perineurial cells; Epi, Epineurial fibroblasts; SC, Schwann cells; nmSC, non-myelinating Schwann cells; mSC, Myelinating Schwann cells; EC, Endothelial cells; EpiEC, Epineurial endothelial cells; EndoEC, Endoneurial endothelial cells; lEC, Lymphatic endothelial cells; Peri, Pericytes; VSM, Vascular smooth muscle cells; VSM/P, Vascular smooth muscle cells and Pericytes. IM, Immune cells. Neutro, Neutrophils; Macro, Macrophages; T/NK/M, T, NK and mast cells.

CellPhoneDB ranks interactions based on the proportion of potentially interacting receptor ligand pairs with significant *p*-values across the cell clusters. In total, we identified 298 significant ligand-receptor interactions in intact nerves (Figure 11A). The largest number of interactions occur between epineurial fibroblasts, perineurial cells and endoneurial fibroblasts in the intact nerve. Subcluster 2 and 7 of epineurial fibroblasts have the highest number of potential ligand-receptor interactions (Figure 11B). Myelinating Schwann cells are known to become quiescent in adult nerves (Tikoo et al., 2000; Stierli et al., 2018) and our CellPhoneDB analysis also revealed that myelinating Schwann cells have the lowest number of ligand-receptor interactions compared with any other cell types in the intact nerve (Figure 11B). All significant ligand-receptor interactions in intact nerves were shown in Supplementary Table 6.

In total, we identified 213 significant ligand-receptor interactions in the distal nerve at day 3 post-injury (Figure 11C). The most significant interactions occur between fibroblasts, macrophages, Schwann cells and cells associated with the blood supply system (Figure 11D). Epineurial fibroblasts and perineurial cells have the highest number of ligand-receptor interactions with cells associated with the blood supply system at day 3 post-injury such as Vegfa-Ephb2, Vegfa-Flt1, and Vegfa-Nrp interactions between epineurial fibroblasts and endothelial cells (Supplementary Table 7). This is the key stage of blood vessel regeneration following peripheral nerve injury (Cattin et al., 2015), indicating that signals from epineurial fibroblasts and perineurial cells may contribute significantly to early blood vessel regeneration. In contrast to the intact nerves, Schwann cells in the distal nerve increase the number of interactions with fibroblasts, macrophages and cells associated with the blood supply system (Figure 11D). At day 3 post-injury, Schwann cells have the highest number of interactions with macrophages. Among identified ligands, Tnfα, Il1β, Csf1, and Tgfβ1 are important signals secreted by macrophages to interact with Schwann cells (Supplementary Table 7), indicating that cytokines secreted by macrophages could be important signals to activate Schwann cells at this stage of Wallerian degeneration. At day 3 post-injury, neutrophils potentially also strongly communicate with macrophages (Figure 11D). Examination of ligands revealed that Tnfα, Il1β, Ccl3, and Ccl4 are important signals secreted by neutrophils to activate resident macrophages or attract monocytes from the circulating blood (Supplementary Table 7). T cells, B cells, NK cells and mast cells have a low number of interactions with other cell types compared to fibroblasts, macrophages, Schwann cells and cells associated with the blood supply system. All significant ligand-receptor interactions in the distal nerve at day 3 post-injury are shown in Supplementary Table 7.

In total, we identified 260 significant ligand-receptor interactions in the distal nerve at day 9 post-injury (Figure 11E). At day 9 post-injury, the most significant interactions occur between subgroups of fibroblasts. The highest number of interactions happened between epineurial fibroblasts with perineurial cells, and perineurial cells with differentiating fibroblasts (Figure 11F). Examining the ligand-receptors pairs showed that Collagen-Integrin, Ephrin-Eph and Fgf-Fgfr interactions are the most important signaling pathways between the subgroups of fibroblasts (Supplementary Table 8). Neutrophils, T cells, B cells, NK cells and mast cells have a low number of interactions with other cell types at day 9 post-injury. All significant ligand-receptor interactions in the distal nerve at day 9 post-injury are shown in Supplementary Table 8.

## Discussion

scRNA-seq allows the cellular composition of complex tissues to be defined in an unbiased fashion (Haque et al., 2017; Hwang et al., 2018). Carr et al. reported the first use of scRNA-seq on the transected mouse sciatic nerve at day 9 post-injury with a focus on studying the function of mesenchymal cells in peripheral nerve regeneration (Carr et al., 2019). One year later, the same group, Toma et al., reported their combined scRNA-seq data at both day 3 and day 9 post-injury for transected mouse sciatic nerve focussing on the study of secreted signals in the distal nerve stump (Toma et al., 2020). In the same year, Wolbert et al. also reported their scRNA-seq data analysis for intact mouse sciatic nerve and the brachial nerve plexus (Wolbert et al., 2020). However, differences exist for the analysis of these three available scRNA-seq data sets in terms of identified cell clusters and suitable marker genes for cell cluster identification. Wolbert et al. (2020) identified the lymphatic endothelial cell cluster that had not been reported by Carr et al. (2019) and Toma et al. (2020). Wolbert et al. (2020) reported a novel endothelial cell cluster in the intact nerve, which shares a similar gene expression profile to perineurial cells as identified by Carr et al. (2019) and Toma et al. (2020). Furthermore, endoneurial fibroblasts were named as nmSCs in Wolbert et al.'s analysis due to endoneurial fibroblasts expressing Ngfr, and therefore they suggested marker genes for endoneurial fibroblasts as marker genes for nmSC, for example Somc2 and Apod (Wolbert et al., 2020). Moreover, neutrophils, a previously well-characterized cell population in the injured nerves by both immunostaining and flow cytometry (Cattin et al., 2015; Lindborg et al., 2017), had not previously been identified in these scRNA-seq data analyses (Carr et al., 2019; Toma et al., 2020). Therefore, we re-analyzed these data sets and provided our rationale for the identification of cell clusters in both intact and injured mouse scitic nerve.

The intact nerve trunk contains both myelinating Schwann cells (mSCs) and non-myelinating Schwann cells (nmSCs). mSCs wrap around and insulate large diameter axons of motor and sensory neurons, forming a myelin sheath, which allows conduction of rapid action potentials. nmSCs ensheath several small diameter axons forming Remak bundles. In adult nerves, mSCs and nmSCs can be easily distinguished by their morphology or myelin protein immunostaining. In our scRNA-seq data analysis, mSCs (cluster 10, Figure 1A) and nmSCs (cluster 1, Figure 1A) could be easily separated into two distinct clusters in intact nerves. However, injury to a peripheral nerve results in both mSCs and nmSCs in the distal nerve stump being rapidly reprogramed into a repair cell phenotype (Jessen and Mirsky, 2016, 2019). The clearance of myelin proteins, the cell morphology change and the down-regulation of classic mSC and nmSC markers make it difficult to distinguish the mSC and nmSC lineage in the distal nerve stump. This might explain why in our analysis, all Schwann cells (SC) cluster into one group, indicating that both mSCs and nmSCs dedifferentiate into more similar phenotypes in the distal nerve following peripheral nerve injury.

S100b has been frequently used as an immunostaining marker to label Schwann cells in both intact and injured nerves. Ngfr/p75 labeling is more frequently used to label Schwann cells in the injured nerves. However, a study has indicated that endoneurial fibroblasts, which makes up 12.5% of the cells within an intact peripheral nerve, also express Ngfr/p75 (Stierli et al., 2018). Recent scRNA-seq data analysis has used Ngfr/p75, S100b, Erbb3, and Sox10 to identify Schwann cell clusters in intact and injured nerves (Carr et al., 2019; Toma et al., 2020; Wolbert et al., 2020). We first tested these markers but found that S100b is not an ideal marker in scRNA-seq data analysis to identify SCs in injured nerves although it can be used as a marker to identify both mSCs and nmSCs clusters in intact nerve (Supplementary Figure 7). In contrast, Ngfr is not a suitable marker to identify both mSCs and nmSCs in intact nerves while Erbb3 is not the best marker to identify mSC in intact nerves (Supplementary Figure 7). Through our analysis, we show that Sox10 and Plp1 are more suitable marker genes in scRNA-seq data analysis to identify Schwann cell clusters in both intact and injured nerves (Supplementary Figure 8). Sox10 is a transcription factor required to specify the Schwann cell lineage (Finzsch et al., 2010). Although Proteolipid protein 1 (PLP1) is a form of myelin proteolipid protein (PLP), transgenic mice expressing green fluorescence protein under the PLP promoter labels both mSCs and nmSCs in intact nerves as well as all SCs in the injured nerves (Mallon et al., 2002; Chen et al., 2019a; Dun et al., 2019). For identifying mSCs in intact nerves, Mbp, Mpz, Mag and Egr2 are all good marker genes (Supplementary Figure 8). Testing marker genes used for identifying nmSCs in the intact nerve, we found that Cdh2 and L1cam are good marker genes (Supplementary Figure 8). Previously, Wolbert et al. have suggested Smoc2 and Apod as nmSC markers (Wolbert et al., 2020). We also analyzed their data set, our analysis indicated that the cluster of endoneurial fibroblasts has been named as nmSCs due to endoneurial fibroblasts expressing Ngfr/p75 (Supplementary Figure 7). In agreement with Carr et al's. findings (Carr et al., 2019), we showed that Smoc2 and Apod have their highest expression in endoneurial fibroblasts (Supplementary Figure 9).

Fibroblasts are another important cell type in peripheral nerves; they are the most abundant cells in the endoneurium, perineurium and epineurium and provide structural support, regional separation and protection of nerve fibers from damage (Parrinello et al., 2010; Stierli et al., 2018; Carr et al., 2019; Diaz-Flores et al., 2020). They have mesenchymal cell properties and play an important role in nerve repair following injury (Parrinello et al., 2010; Carr et al., 2019). We tested marker genes for fibroblasts reported in previous mouse sciatic nerve scRNA-seq studies including: Sfrp4, Pi16, Dpt, Gsn, Col1a1, Col1a2, Col3a1, Col14a1, Clec3b, Cygb, Prrx1, and Aebp1 (Carr et al., 2019; Diaz-Flores et al., 2020; Toma et al., 2020; Wolbert et al., 2020). We found that some of these genes are not fibroblast-specific and that while some of them were fibroblast specific, they only label one sub-population of epineurial, perineurial or endoneurial fibroblasts. Particularly, most of the genes show up-regulation in other cells types in response to injury. We therefore screened DEGs and revealed that Mfap5 and Serpinf1 are better marker genes to identify fibroblast clusters in both intact and injured nerve (Supplementary Figure 10). Studies have shown that microfibrillar-associated protein 5 (Mfap5), is a fibroblast derived factor, which can promote tumor cell epithelial-mesenchymal transition, migration and metastasis (Valenzi et al., 2019; Chen et al., 2020). Examination of gene expression in mesenchymal cells of the lung by single-cell analysis also found that lung fibroblasts express high levels of Mfap5 (Valenzi et al., 2019). Serpinf1 encodes the pigment epithelium-derived factor (PEDF) and is secreted by fibroblasts. Homozygous mutations in Serpinf1 cause deficiency of PEDF, which leads to osteogenesis imperfecta (Al-Jallad et al., 2014). Similar to the function of Mfap5, loss of PEDF in cancer cells is associated with poor prognosis and metastasis (Nwani et al., 2016). Using these analyses, we identified that cell cluster 0, 2, 6, 7 and 11 in intact nerve are fibroblasts (Figure 1A), cell cluster 0, 2, 3, and 5 in post-injury day 3 nerves are fibroblasts (Figure 2A), and cell cluster 0, 4, 7, and 10 in post-injury day 9 nerves are fibroblasts (Figure 3A).

According to the anatomical location, peripheral nerve fibroblasts could be divided into endoneurial, perineurial and epineurial fibroblasts (Osawa and Ide, 1986; Pina-Oviedo and Ortiz-Hidalgo, 2008). Endoneurial fibroblasts are spindle-shaped cells with long processes making contact with other cell types in the endoneurium, they are present between nerve fibers and compose 12.5% of all endoneurial cells (Stierli et al., 2018; Carr et al., 2019; Diaz-Flores et al., 2020). Endoneurial fibroblasts have also been named as tactocytes (Stierli et al., 2018), endoneurial mesenchymal cells (Carr et al., 2019) and endoneurial telocytes (Diaz-Flores et al., 2020). They express marker genes Sox9 and Osr2, they also up-regulate Wif1 in response to nerve injury (Carr et al., 2019; Toma et al., 2020). Using these reported marker genes together with DEGs in our analysis (Supplementary Tables 1–3), we found that Sox9, Osr2, Wif1, Abca9, Cdkn2a, Cdkn2b, and Plxdc1 are all good marker genes for the identification of endoneurial fibroblasts (Supplementary Figure 11). Our analysis revealed that cluster 0 in intact nerves, cluster 2 in nerves at 3 days post-injury and cluster 4 in nerves at 9 days post-injury are all endoneurial fibroblasts (Figures 1–3).

Axons targeting the same anatomical location within the nerve are bundled together into fascicles by a protective sheath known as the perineurium (Pina-Oviedo and Ortiz-Hidalgo, 2008). Fibroblasts in the perineurium have long been named as perineurial cells (Strauss and Cohen, 1981; Theaker and Fletcher, 1989; Inokuchi et al., 1991; Schroder et al., 1993; Weis et al., 1994; Kucenas, 2015). Perineurial cells are the first cell type to migrate into the nerve bridge following peripheral nerve transection injury and form a perineurial tube to control the trajectory and migration of other cells (Schroder et al., 1993; Weis et al., 1994; Min et al., 2020). Perineurial cells express marker genes such as Slc2a1/Glut1, Lypd2, Sfrp5, Ntn4, Msln, Ntng1, the tight junction genes Tjp1/ZO-1, the desmosome protein Perp, and integrins Itgb4 and Itga6 (Theaker and Fletcher, 1989; Kucenas, 2015; Carr et al., 2019; Toma et al., 2020; Wolbert et al., 2020). We used these marker genes (Supplementary Figure 11) and identify perineurial cells in intact nerve (cluster 11) as well as post-injury day 9 (cluster 10) nerves (Figures 1A, 3A). However, perineurial cells were clustered into the cluster of differentiating fibroblasts for nerves at 3 days post-injury due to the low number of perineurial cells (Figure 2A). We also performed the integrated data analysis and perineurial cells could be visualized as an individual cluster at day 3 post injury with integrated data analysis (Figure 10B). Our analysis showed that that perineurial cells also share some gene signatures with endothelial cells such as Moxd1, Ntng1, Lypd2, Krt19, and Dleu7 (Supplementary Tables 1–3), which resulted in perineurial cells being identified as an additional cluster of endothelial cells in a recently reported scRNA-seq data analysis (Wolbert et al., 2020).

The epineurium is the outermost layer of connective tissue surrounding and protecting nerve fibers. It contains not only fibroblasts but also immune cells, adipocytes and blood vessel associated cells (Osawa and Ide, 1986). Epineurial fibroblasts highly express Sfrp2, Adamts5, Pcolce2, Clec3b, Pi16, Ly6c1, Dpt, Dpp4, Gsn, Comp, and Sfrp4 (Carr et al., 2019; Toma et al., 2020; Wolbert et al., 2020). Using these marker genes, we identify cluster 2, 6, and 7 as epineurial fibroblasts in intact nerves, cluster 3 and 5 as epineurial fibroblasts in post-injury day 3 nerves, and cluster 7 as epineurial fibroblasts in post-injury day 9 nerves (Supplementary Figure 10). We used a resolution of 0.75 for cell clustering in the intact nerves in order to label the cell cluster of lymphatic endothelial cells. This has resulted in the separation of epineurial fibroblasts into three sub-clusters in intact nerves. Similar to cluster 0 of endoneurial fibroblasts in intact nerves, cluster 2 and cluster 7 epineurial fibroblasts express high levels of Pdgfr2 (Supplementary Figure 3) which is a mesenchymal cell marker for epi- and endoneurial mesenchymal-like fibroblasts (Carr et al., 2019). Indeed, in Pdgfra-EGFP mice, epineurial sheaths contain 60.5 ± 6.0% GFP positive mesenchymal cells (Carr et al., 2019; Toma et al., 2020). In response to injury, fibroblasts in the distal nerve undergo differentiation and differentiating nerve fibroblasts could be identified using differentiating marker genes Dlk1, Mest, Cilp, Tnc, Plagl1, and Ptn (Carr et al., 2019; Toma et al., 2020). Using these marker genes, we identified the largest fibroblast cluster (cluster 0) in both post-injury day 3 and post-injury day 9 nerves as differentiating fibroblasts (Supplementary Figure 4).

We use Ptprc/CD45 and Cd52 as general marker genes to identify immune cells in intact and injured nerves (Carr et al., 2019; Wolbert et al., 2020; Ydens et al., 2020). The number of immune cells in the intact nerve is low in data set GSE147285 and all immune cells have been clustered into one cluster (cluster 12, Figure 1) as revealed by Ptprc/CD45 and Cd52 expression (Figure 1A and Supplementary Figure 5). Due to the high number of cells in data set GSE142541 (Wolbert et al., 2020), immune cells could be clustered to B cells (cluster 13 in Figure 6A), epineurial macrophages (cluster 15 in Figure 6A), endoneurial macrophages (cluster 16 in Figure 6A), T cells (cluster 14, 19 and 21 in Figure 6A) and NK cells (cluster 22 in Figure 6A). Previous studies have shown that resident macrophages are the major immune cells in the intact nerve and compose 8–9% cells of the intact mouse sciatic nerve (Stierli et al., 2018; Amann and Prinz, 2020). However, this analysis revealed that the intact mouse peripheral nerves also contain a large number of T cells. In the intact nerve, macrophage express genes Aif1/Iba1, Cd68, and Mrc1/Cd206 (Supplementary Figure 12) and these marker genes label two sub-cluster of macrophages (cluster 15 and 16 in Figure 4A). Macrophages in cluster 15 express Retnla and Clec10a and they are epineurial macrophages (Ydens et al., 2020). Thus, macrophages in cluster 16 are resident macrophages.

Following peripheral nerve injury, a large number of immune cells infiltrate the distal nerve (Gaudet et al., 2011). Ptprc/CD45 and Cd52 label four clusters of cells (cluster 1, 7, 9, and 10) in post-injury day 3 nerves, and label six cell clusters (cluster 2, 5, 8, 9, 11, and 12) in post-injury day 9 nerves (Supplementary Figure 5). Immune cells can be divided into myeloid cell lineages and the lymphoid cell lineage. Myeloid cells include macrophages, neutrophils and mast cells, and can be identified with myeloid marker genes Lyz2, Ccl6, and Lyz1 (Carr et al., 2019; Amann and Prinz, 2020; Kolter et al., 2020; Toma et al., 2020; Wolbert et al., 2020; Ydens et al., 2020). Lymphoid cells include B cells, T cells and NK cells, they can be identified with lymphoid marker genes Ptprcap and Trbc2 (Carr et al., 2019; Toma et al., 2020; Wolbert et al., 2020; Ydens et al., 2020). These markers genes were initially used to distinguish myeloid cells from lymphoid cells in the immune cell clusters (Supplementary Figure 13).

Peripheral nerve injury results in rapid resident macrophage activation and infiltration of a large number of bone marrow derived macrophages into the distal nerve (Dun et al., 2019; Zigmond and Echevarria, 2019; Kolter et al., 2020). Currently, there are no clear markers to distinguish activated resident macrophages from infiltrated macrophages in injured nerves (Amann and Prinz, 2020; Kolter et al., 2020). A recent publication using both Csf1r-ECFP and Cx3cr1-EGFP mice demonstrated that resident macrophages have distinct function and morphological difference from recruited macrophages (Boissonnas et al., 2020). Macrophages in the distal nerve could be identified with macrophage marker genes Aif1/Iba1, Cd68, and Mrc1/Cd206. These marker genes label cluster 1 in day 3 post-injury nerves and cluster 2 in day 9 post-injury nerves (Supplementary Figure 12).

We used marker genes Cma1, Mcpt4, Mcpt1, and Kit to identify mast cells as suggested in recent scRNA-seq data analysis in mouse sciatic nerve samples (Carr et al., 2019; Toma et al., 2020; Wolbert et al., 2020). In our analysis, mast cells clustered together with all immune cells (cluster 12, Figure 1A) in intact nerve and were also clustered with T/NK cells (cluster 7, Figure 2A) in day 3 post-injury nerves. However, t-SNE visualization analysis revealed that mast cells form distinct sub-clusters (indicated by a red circle in Supplementary Figure 14) both in intact nerves and day 3 post-injury nerves although increasing the clustering resolution in our analysis we were unable to further separate them as an individual cluster. The low abundance of mast cells in intact and day 3 post-injury nerves could be the reason that they grouped into a single cluster with other immune cells as mast cells clustered separately (cluster 12, Figure 1A) in day 9 post-injury nerves when more mast cells were apparently present (Supplementary Figure 14).

T cells could be identified by marker genes Cd3e, Cd3g, Cxcr6, and Trac, and NK cells could be identified by marker genes Ncr1, Nkg7, and Klrk1 (Supplementary Figure 15). In day 3 post-injury nerves, T cells and NK cells are found in the same cluster (cluster 7, Figure 2A), but T cells and NK cells cluster separately in day 9 post-injury nerves (cluster 8 for T cells and cluster 11 for NK cells, Figure 3A). Recently, Wolbert et al. showed that B cells could be identified in intact mouse sciatic nerves using marker genes such as Bank1, Ms4a1, Cd19, Cd40, and Cd79a (Wolbert et al., 2020). There is a significant number of B cells in 3 day post-injury nerves and they have been grouped into cluster 10 (Figure 2A and Supplementary Figure 16). There are even more B cells in day 9 post-injury nerves (cluster 5, Figure 3A), indicating a rapid infiltration of B cells into the distal nerve between day 3 and day 9 following injury.

Classic gene markers used in scRNA-seq data analysis to identify ECs include Pecam1/Cd31, Tie1, and Emcn (Zhao et al., 2018; Carr et al., 2019; Kalluri et al., 2019; Toma et al., 2020; Wolbert et al., 2020). We used these markers to identify ECs, which are found in clusters 3, 4 and 5 in intact nerves, cluster 4 in 3 day post-injury nerves, and cluster 1 in 9 day post-injury nerves (Supplementary Figure 5). Recently, Wolbert et al. also identified lymphatic ECs in the intact nerves in their scRNA-seq data analysis using lymphatic EC marker genes Lyve1 and Prox1 (Wolbert et al., 2020). Other scRNA-seq data analysis have shown that lymphatic ECs have distinct gene expression profiles including the expression of Lyve1, Mmrn1, Prox1, and Flt4 (Engelbrecht et al., 2020; Fujimoto et al., 2020). Therefore, we used these marker genes to identify the cluster containing lymphatic ECs. Our analysis showed that Lyve1, Mmrn1, and Flt4 are suitable marker genes to identify the lymphatic EC cluster in intact nerves (Supplementary Figure 1). However, all four distinct EC clusters in the intact nerves have been clustered into just one EC cluster in day 3 and day 9 post-injury nerves (Figures 2A, 3A, Supplementary Figure 17).

Our analysis confirmed that there are three distinct EC subgroups in the intact mouse sciatic nerve: epineurial, endoneurial and lymphatic ECs. However, classic EC markers such as Pecam1/Cd31, Tie1 and Emcn were unable to identify lymphatic ECs (Supplementary Figure 2). In this study, we investigated if there were better marker genes that could label all the ECs in mouse sciatic nerve. Our investigation revealed that Cdh5, another common marker gene for ECs, could label all four EC clusters but Cdh5 could also label perineurial cells in both intact and injured nerves (Supplementary Figure 17). Further analysis indicates that Egfl7 and Ecscr are the better marker genes to identify all types of ECs in mouse sciatic nerve for scRNA-seq data analysis (Supplementary Figure 17). EGFL7 is a highly conserved angiogenic factor in vertebrates. Unlike most secreted angiogenic signaling molecules such as vascular endothelial growth factor and fibroblast growth factor-2, which are mainly expressed by non-endothelial cell types, EGFL7 is almost exclusively expressed and secreted by endothelial cells, it binds to components of the extracellular matrix and acts as a chemoattractant for endothelial cells (Nichol and Stuhlmann, 2012). EGFL7 also regulates the collective migration of endothelial cells and controls their spatial distribution (Schmidt et al., 2007). EGFL7 expression is highest when the endothelium is in an active and proliferating state (Nichol and Stuhlmann, 2012). In contrast, ECSCR is an endothelial cell-specific chemotaxis receptor selectively expressed by endothelial cells; it plays roles in endothelial cell migration, proliferation and promotes angiogenesis (Verma et al., 2010; Kilari et al., 2013). The high expression of EGFL7 and ECSCR in mouse sciatic nerve indicates that EGFL7 and ECSCR could play important roles in peripheral nerve vascular homeostasis and regeneration following injury.

In conclusion, we re-analyzed recently published single-cell RNA sequencing data sets and generated a cellular composition map of the peripheral nerve in homeostasis and regeneration. We identified each cell type using marker genes reported from the literature (Carr et al., 2019; Toma et al., 2020; Wolbert et al., 2020). In addition, DEGs between clusters were used to established suitable marker genes for future single cell transcriptomic analyses for the identification of cell types in intact and injured peripheral nerves. Our analysis also revealed three sub-groups of endothelial cells in the intact nerve and identified the neutrophil cluster in injured nerves, which were not reported in previous studies (Carr et al., 2019; Toma et al., 2020; Wolbert et al., 2020). Identification of these cell clusters will enable us to further study their distinct gene expression profiles for each cell type and the signals that mediate cell-cell communication in intact and injured peripheral nerves. The findings from our analysis could facilitate a better understanding of the cell biology of peripheral nerves in homeostasis, regeneration and disease.

## Data Availability Statement

The original contributions presented in the study are included in the article/supplementary material, further inquiries can be directed to the corresponding author/s.

## Author Contributions

XD designed the research. MB, BC, and XD performed scRNA-seq data analysis. LS performed immunostaining. BC, DP, and XD wrote the paper. All authors contributed to the article and approved the submitted version.

## Conflict of Interest

The authors declare that the research was conducted in the absence of any commercial or financial relationships that could be construed as a potential conflict of interest.
