# Supplementary material for: Single Cell Transcriptome Data Analysis Defines the Heterogeneity of Peripheral Nerve Cells in Homeostasis and Regeneration
Source: Front Cell Neurosci. 2021 Mar 22;15:624826. doi: 10.3389/fncel.2021.624826 (PMC8019921; doi:10.3389/fncel.2021.624826)
Supplement: Supplementary file 1 [file Data_Sheet_1.PDF]

Figure S1

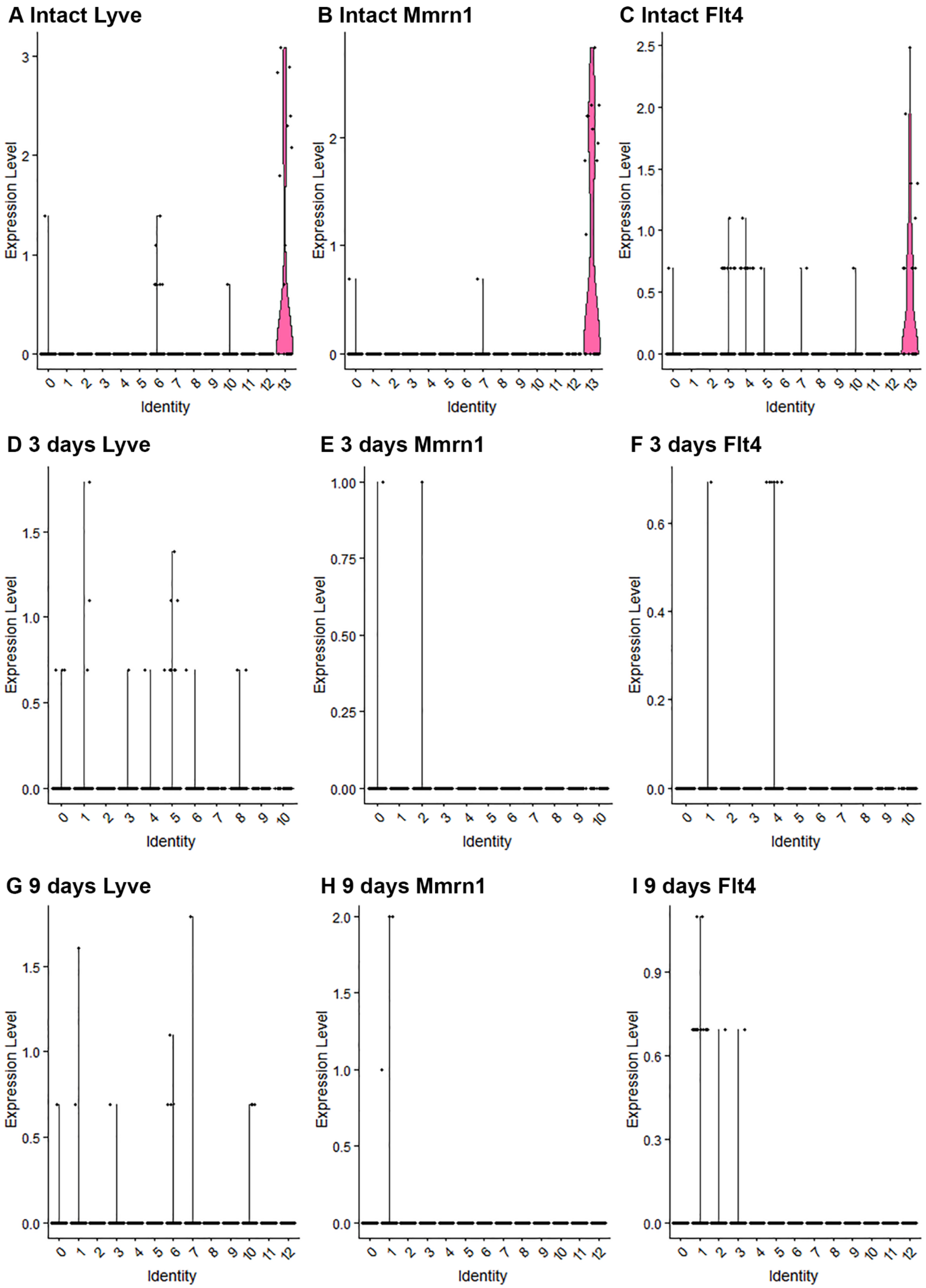
A Intact Lyve
B Intact Mmrn1
C Intact Flt4
D 3 days Lyve
E 3 days Mmrn1
F 3 days Flt4
G 9 days Lyve
H 9 days Mmrn1
I 9 days Flt4
Expression Level
Identity

Figure S2

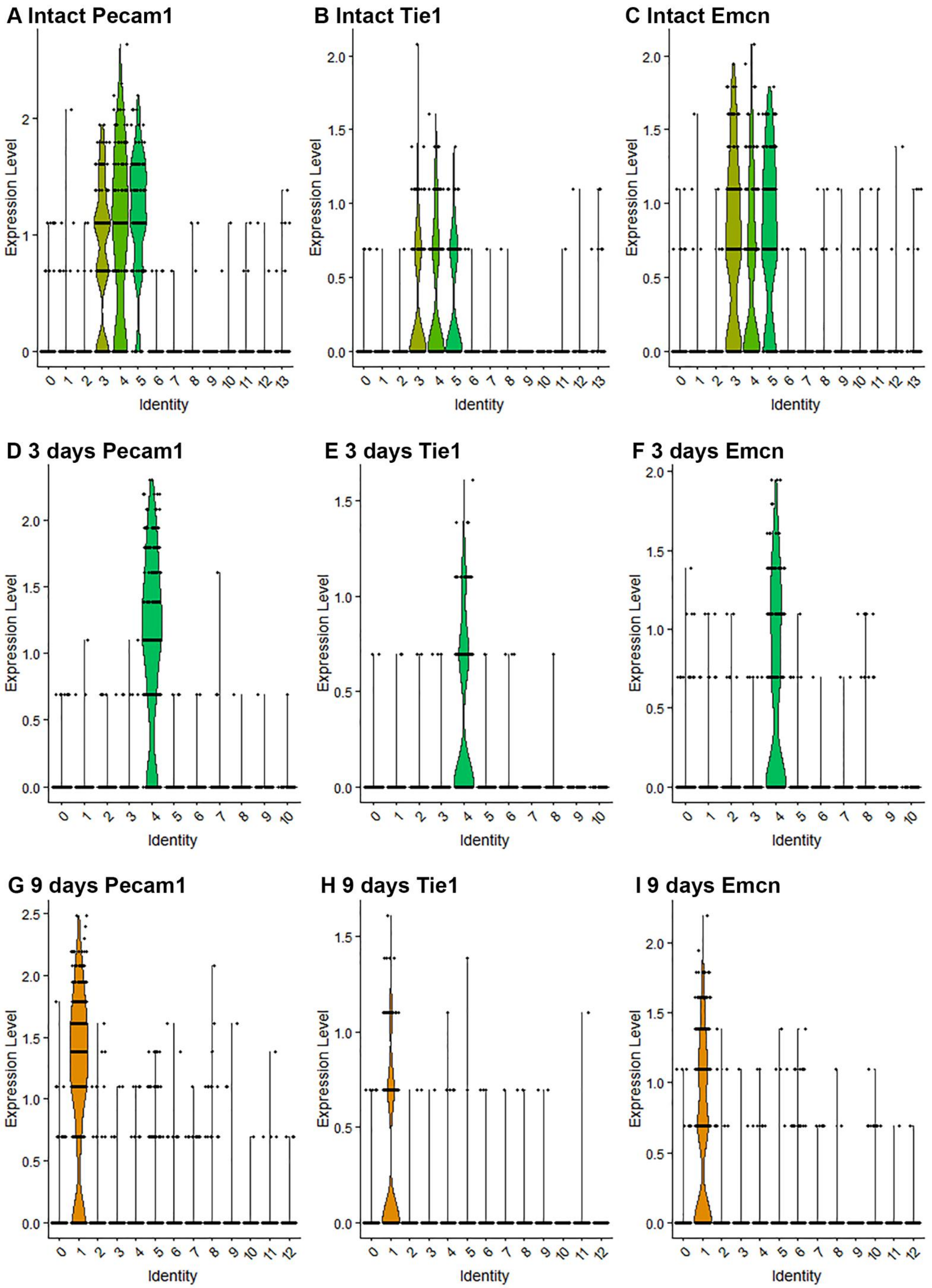

A Intact Pecam1
B Intact Tie1
C Intact Emcn
D 3 days Pecam1
E 3 days Tie1
F 3 days Emcn
G 9 days Pecam1
H 9 days Tie1
I 9 days Emcn
Expression Level
Identity

Figure S3

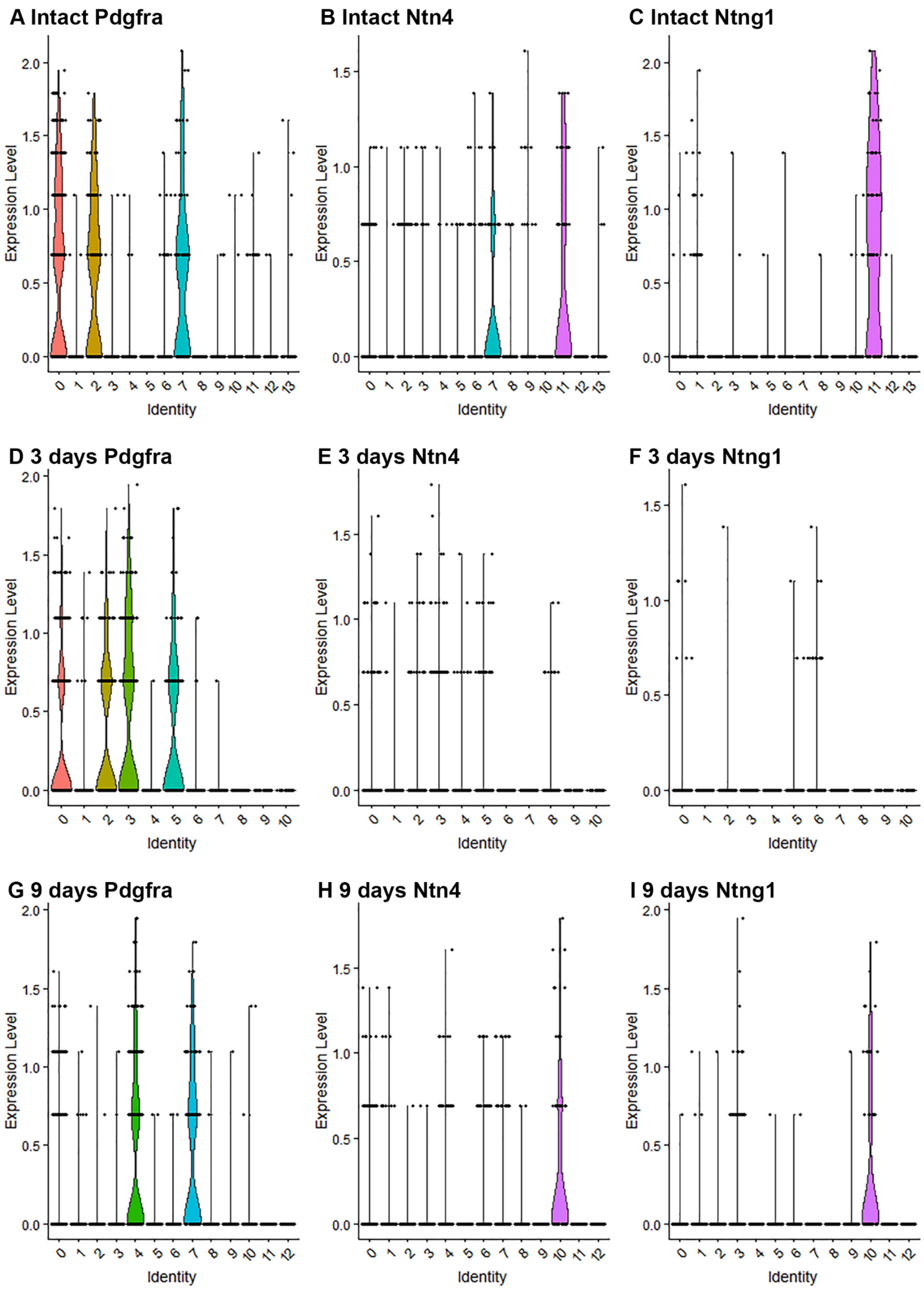

A Intact Pdgfra
B Intact Ntn4
C Intact Ntng1
D 3 days Pdgfra
E 3 days Ntn4
F 3 days Ntng1
G 9 days Pdgfra
H 9 days Ntn4
I 9 days Ntng1
Expression Level
Identity

Figure S4

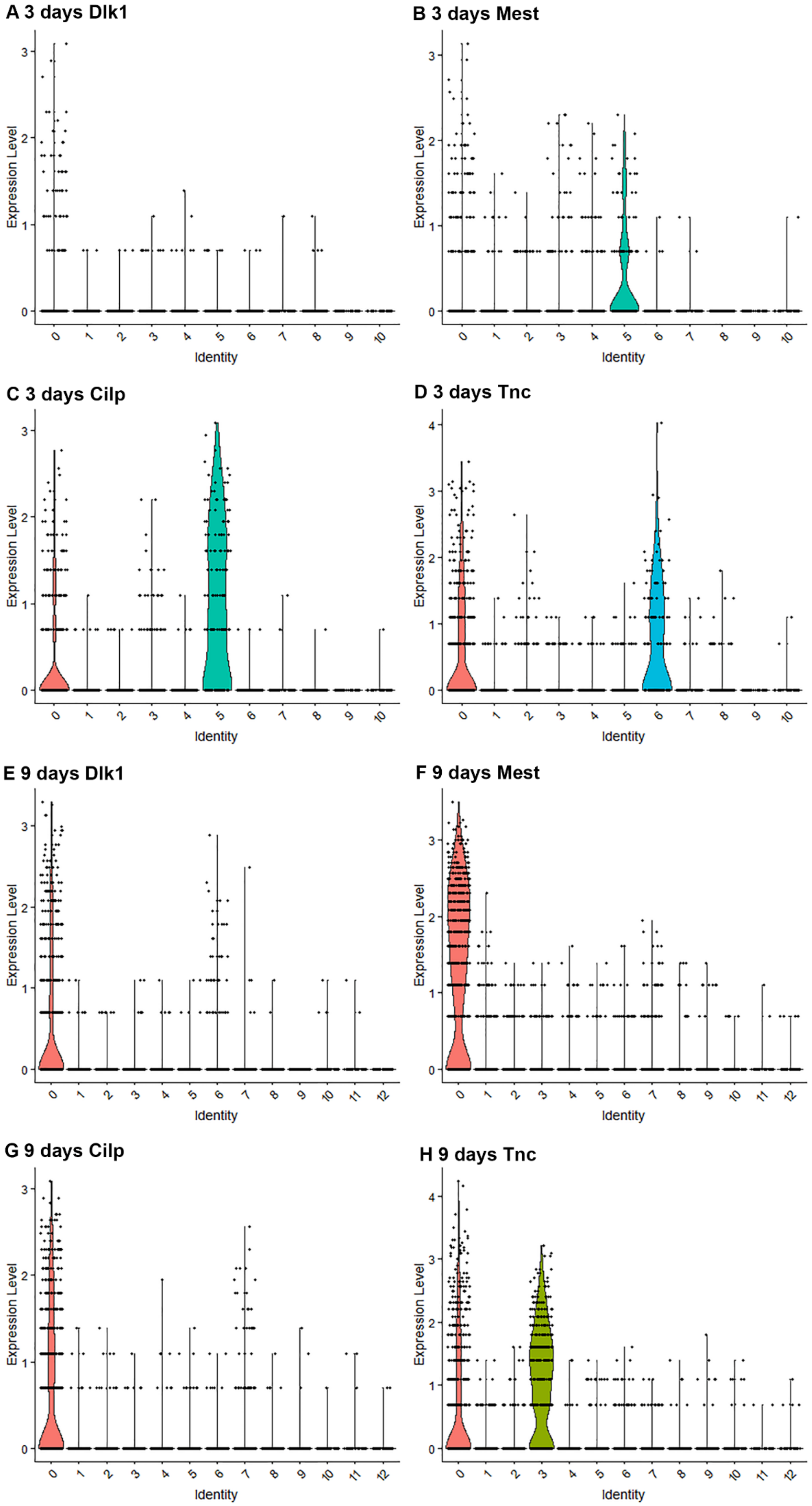

A 3 days Dlk1
B 3 days Mest
C 3 days Cilp
D 3 days Tnc
E 9 days Dlk1
F 9 days Mest
G 9 days Cilp
H 9 days Tnc
Expression Level
Identity

Figure S5

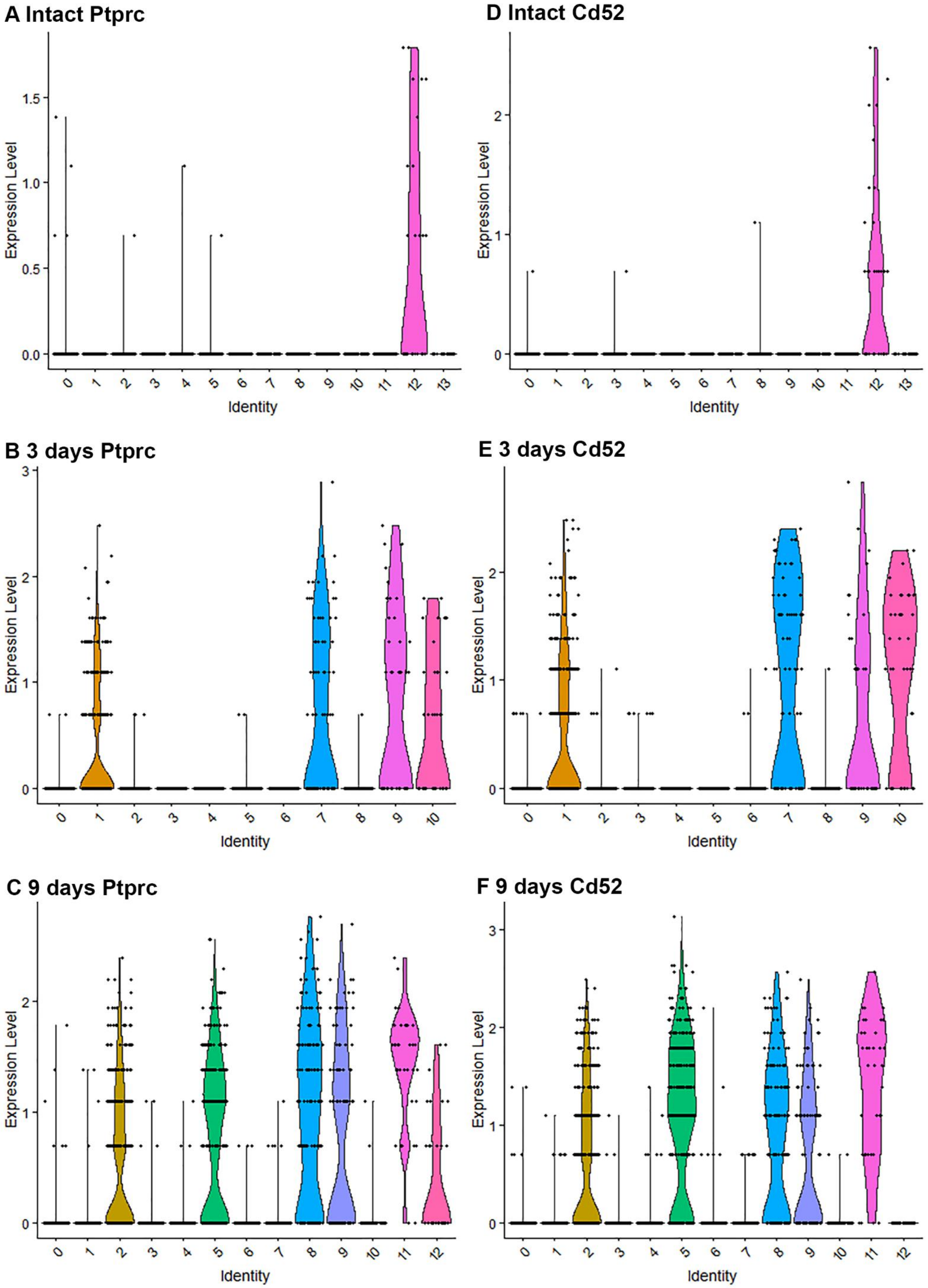
A Intact Ptprc
D Intact Cd52
B 3 days Ptprc
E 3 days Cd52
C 9 days Ptprc
F 9 days Cd52
Expression Level
Identity

Figure S6

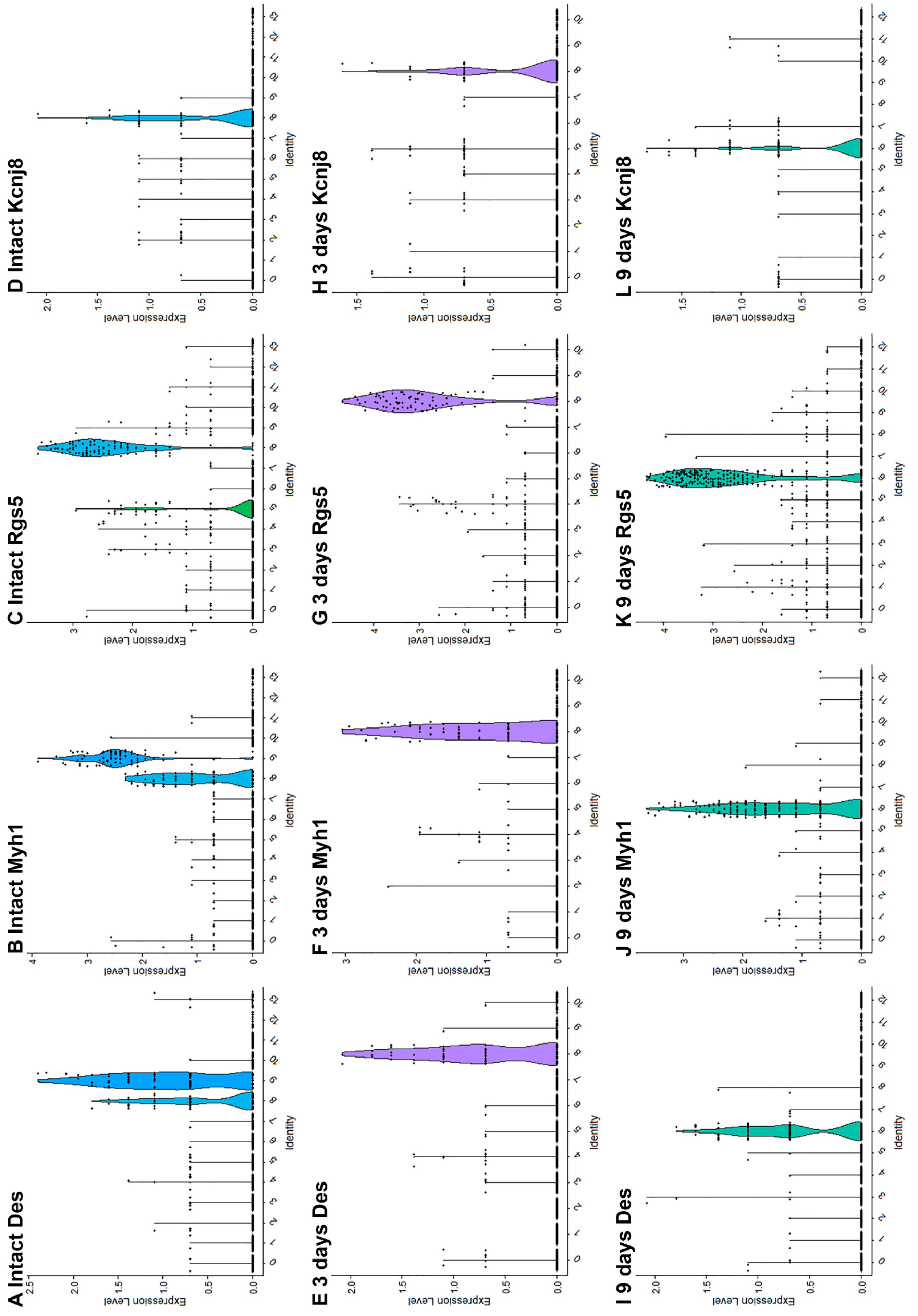

A Intact Des
B Intact Myh1
C Intact Rgs5
D Intact Kcnj8
E 3 days Des
F 3 days Myh1
G 3 days Rgs5
H 3 days Kcnj8
I 9 days Des
J 9 days Myh1
K 9 days Rgs5
L 9 days Kcnj8
Expression Level
Identity

Figure S7

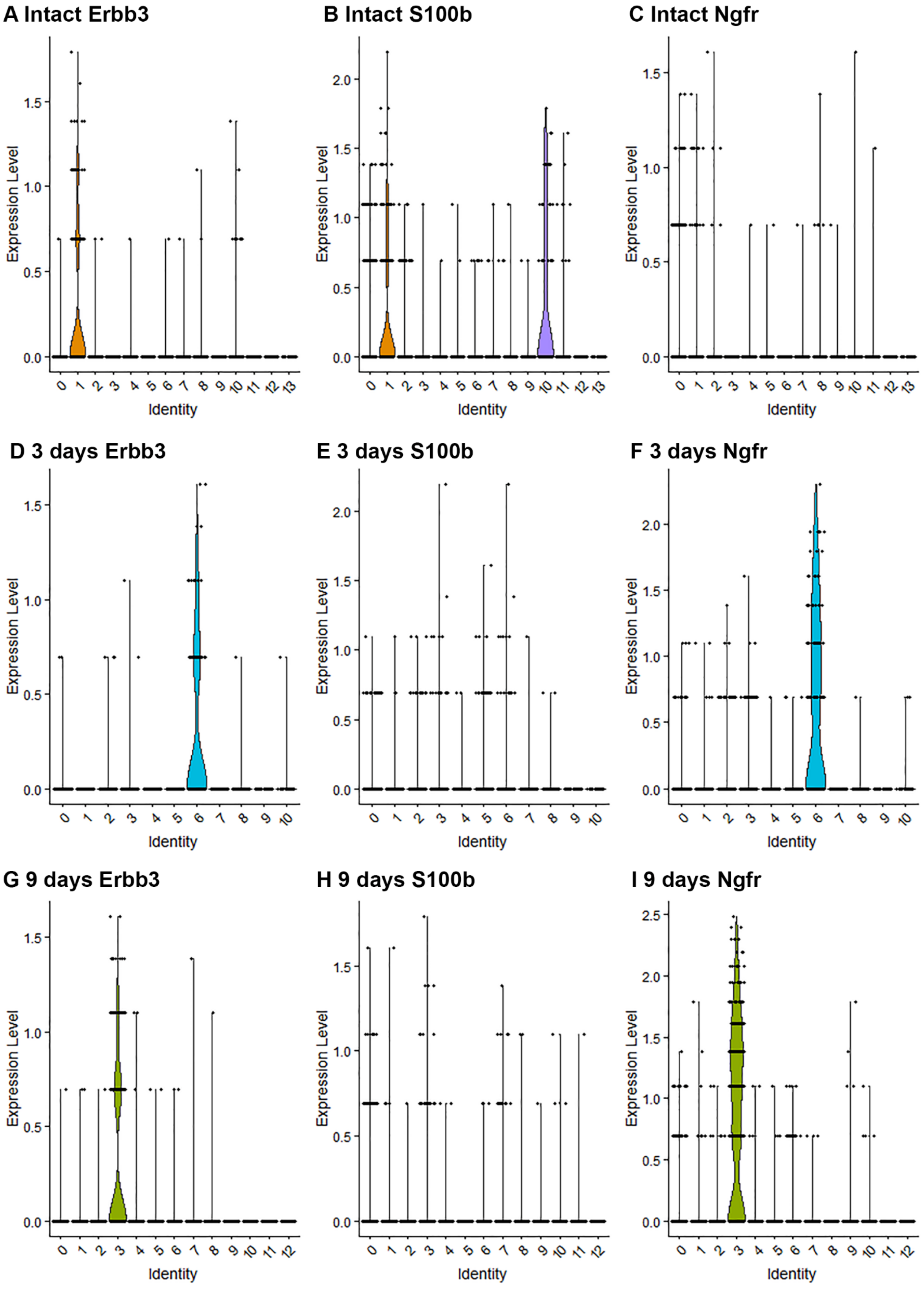
A Intact Erbb3
B Intact S100b
C Intact Ngfr
D 3 days Erbb3
E 3 days S100b
F 3 days Ngfr
G 9 days Erbb3
H 9 days S100b
I 9 days Ngfr
Expression Level
Identity

Figure S8

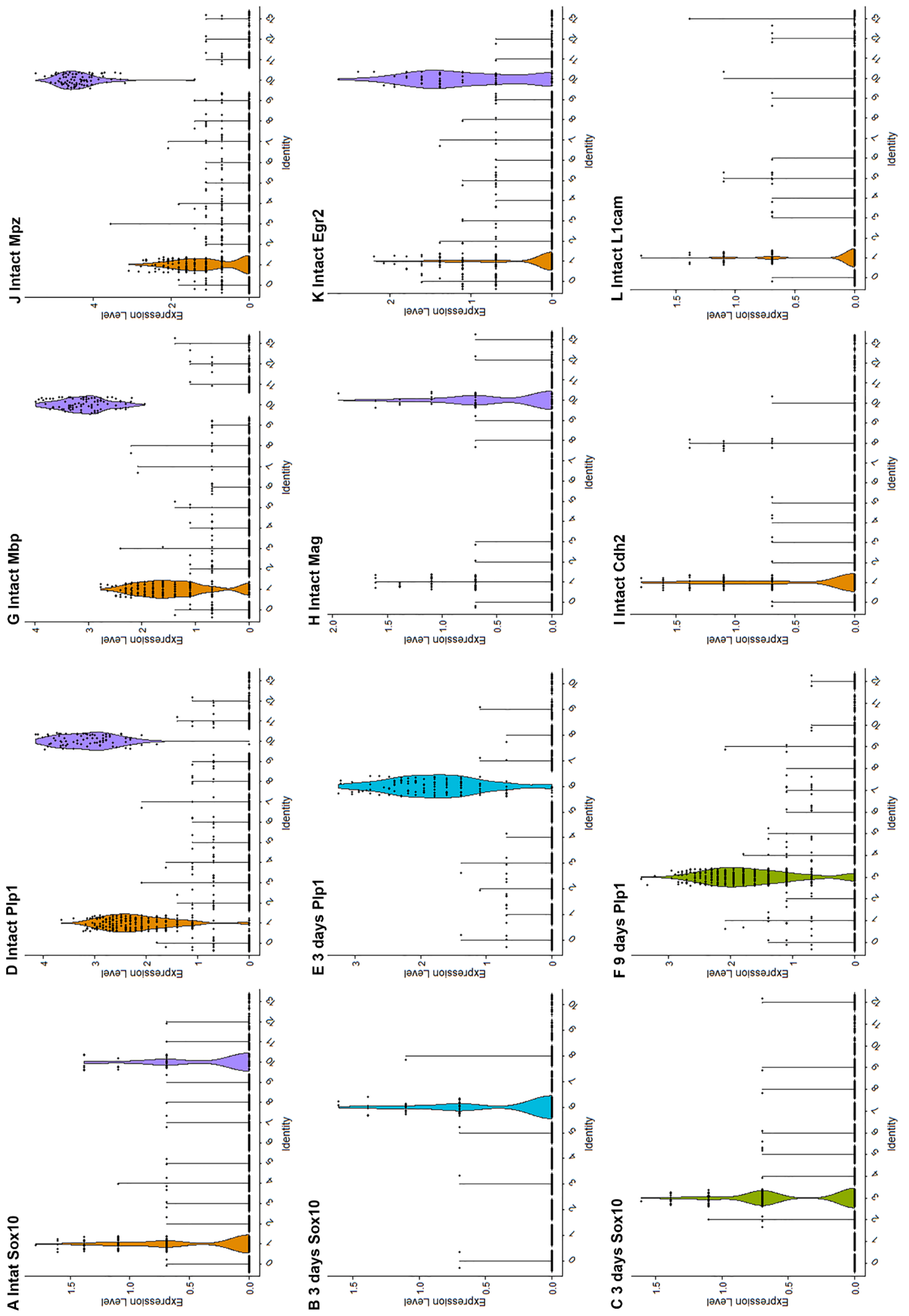

A Intat Sox10
B 3 days Sox10
C 3 days Sox10
D Intact Plp1
E 3 days Plp1
F 9 days Plp1
G Intact Mbp
H Intact Mag
I Intact Cdh2
J Intact Mpz
K Intact Egr2
L Intact L1cam
Expression Level
Identity

Figure S9

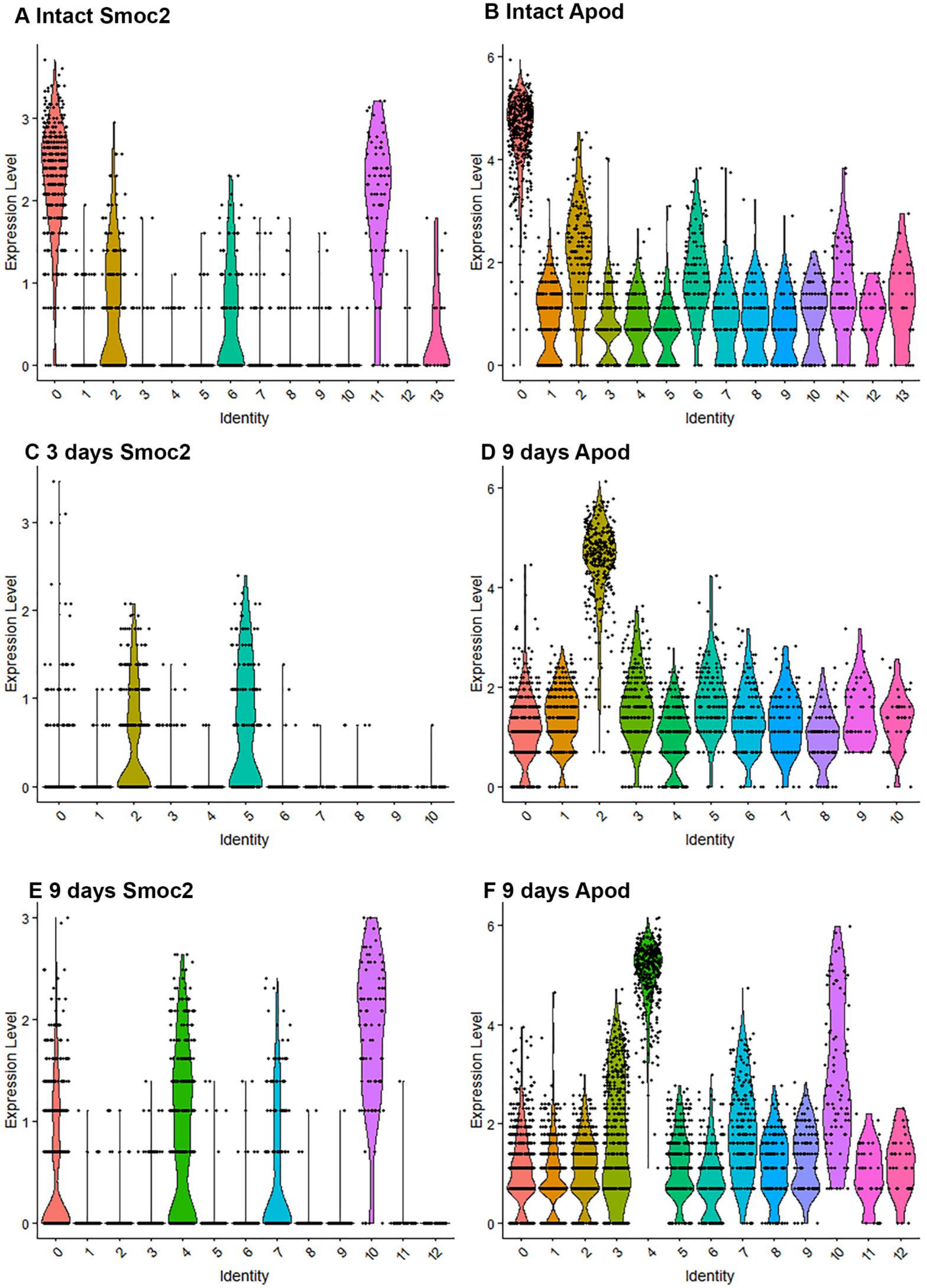
A Intact Smoc2
B Intact Apod
C 3 days Smoc2
D 9 days Apod
E 9 days Smoc2
F 9 days Apod
Expression Level
Identity

Figure S10

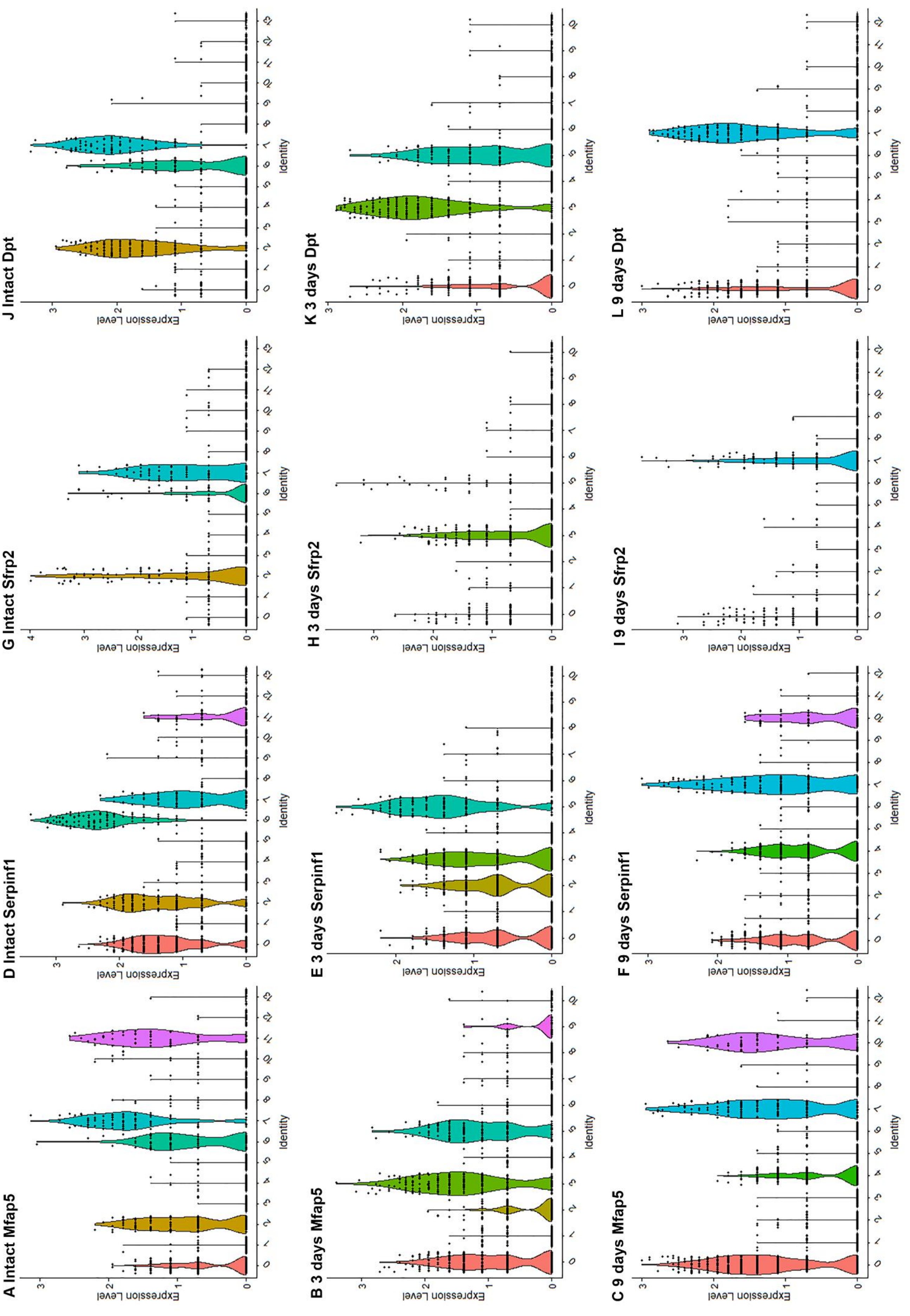
A Intact Mfap5
B 3 days Mfap5
C 9 days Mfap5
D Intact Serpinf1
E 3 days Serpinf1
F 9 days Serpinf1
G Intact Sfrp2
H 3 days Sfrp2
I 9 days Sfrp2
J Intact Dpt
K 3 days Dpt
L 9 days Dpt
Expression Level
Identity

Figure S11

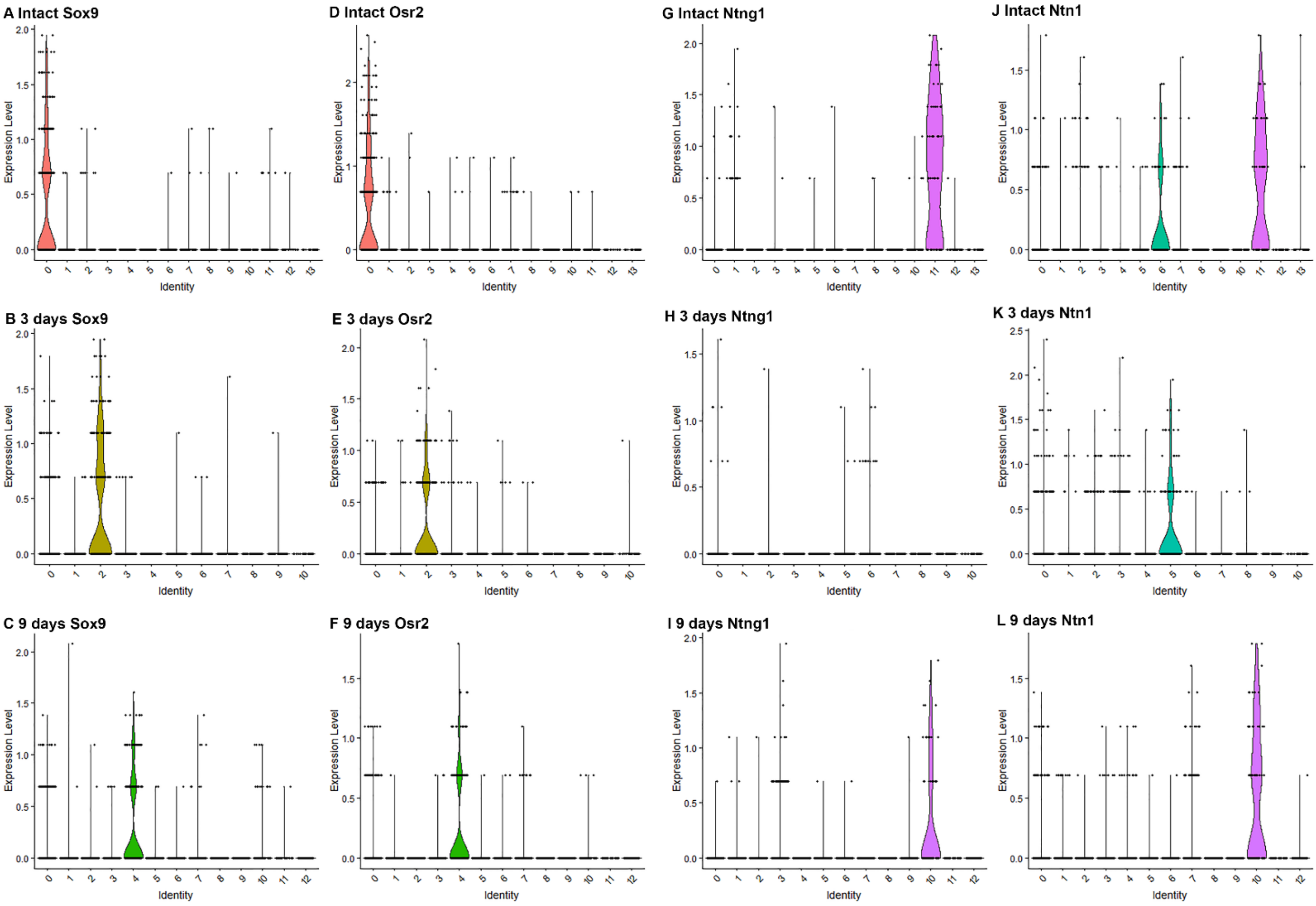
A Intact Sox9
D Intact Osr2
G Intact Ntng1
J Intact Ntn1
B 3 days Sox9
E 3 days Osr2
H 3 days Ntng1
K 3 days Ntn1
C 9 days Sox9
F 9 days Osr2
I 9 days Ntng1
L 9 days Ntn1
Expression Level
Identity

Figure S12

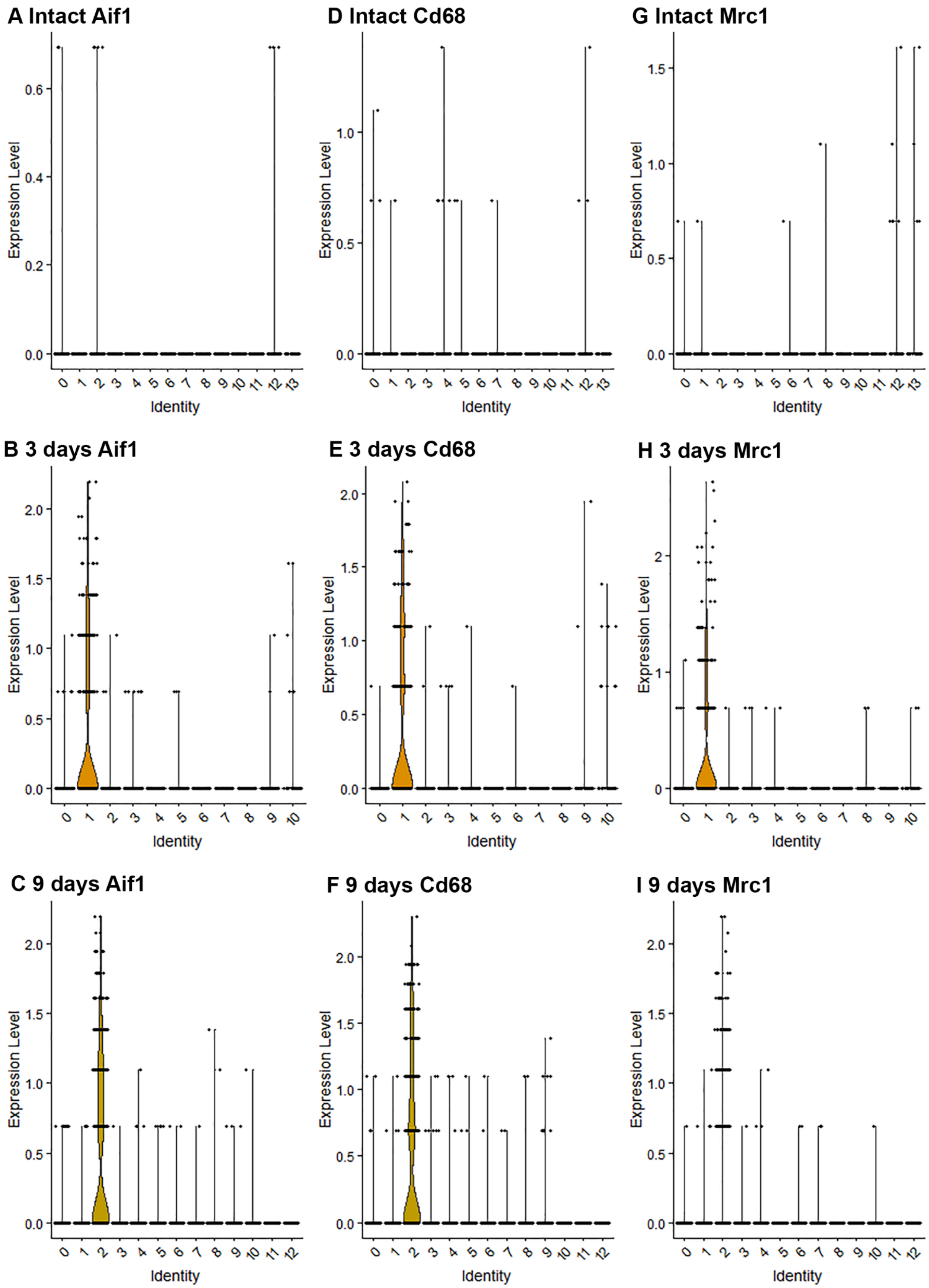

A Intact Aif1
D Intact Cd68
G Intact Mrc1
B 3 days Aif1
E 3 days Cd68
H 3 days Mrc1
C 9 days Aif1
F 9 days Cd68
I 9 days Mrc1
Expression Level
Identity

Figure S13

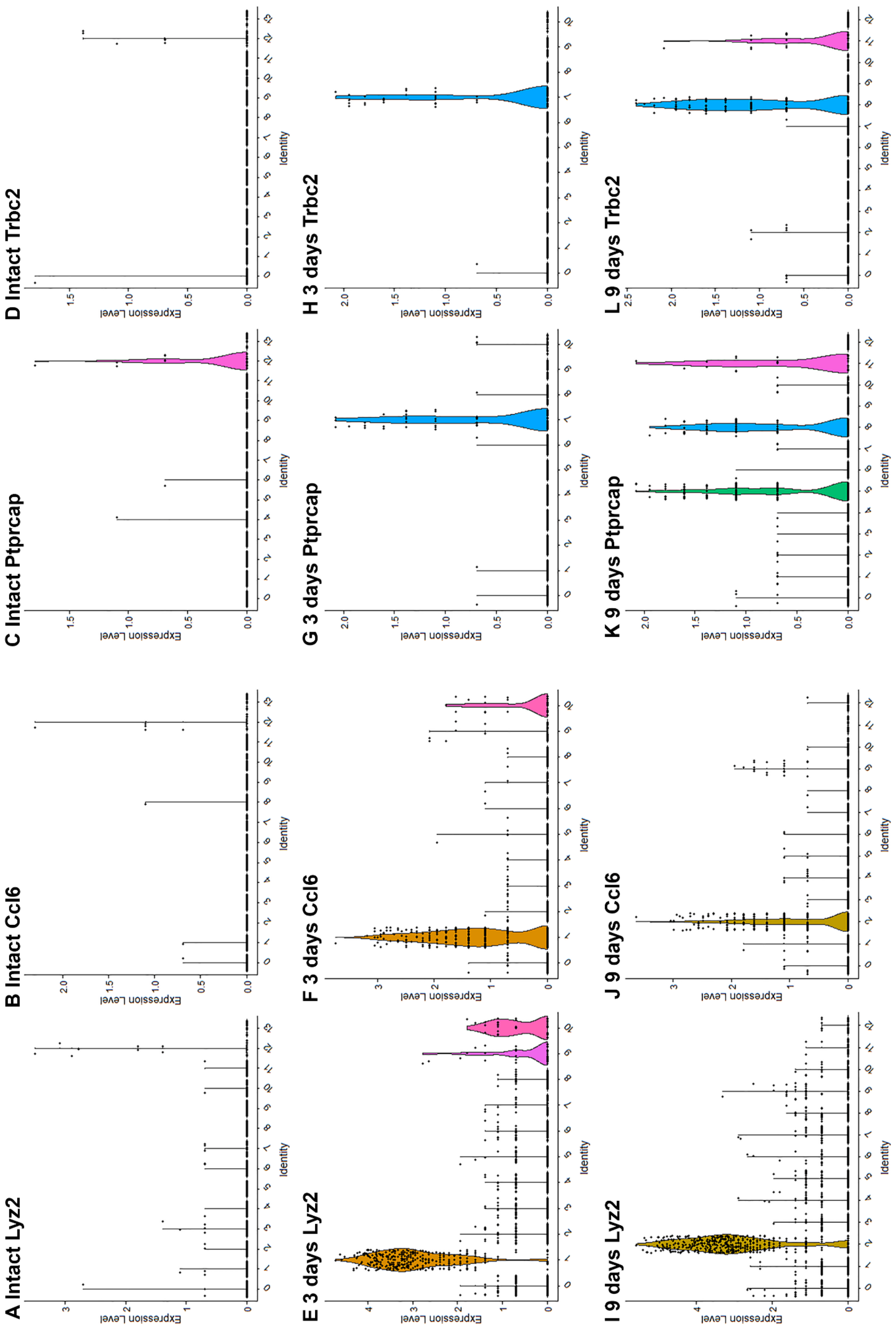
A Intact Lyz2
B Intact Ccl6
C Intact Ptprcap
D Intact Trbc2
E 3 days Lyz2
F 3 days Ccl6
G 3 days Ptprcap
H 3 days Trbc2
I 9 days Lyz2
J 9 days Ccl6
K 9 days Ptprcap
L 9 days Trbc2
Expression Level
Identity

Figure S14

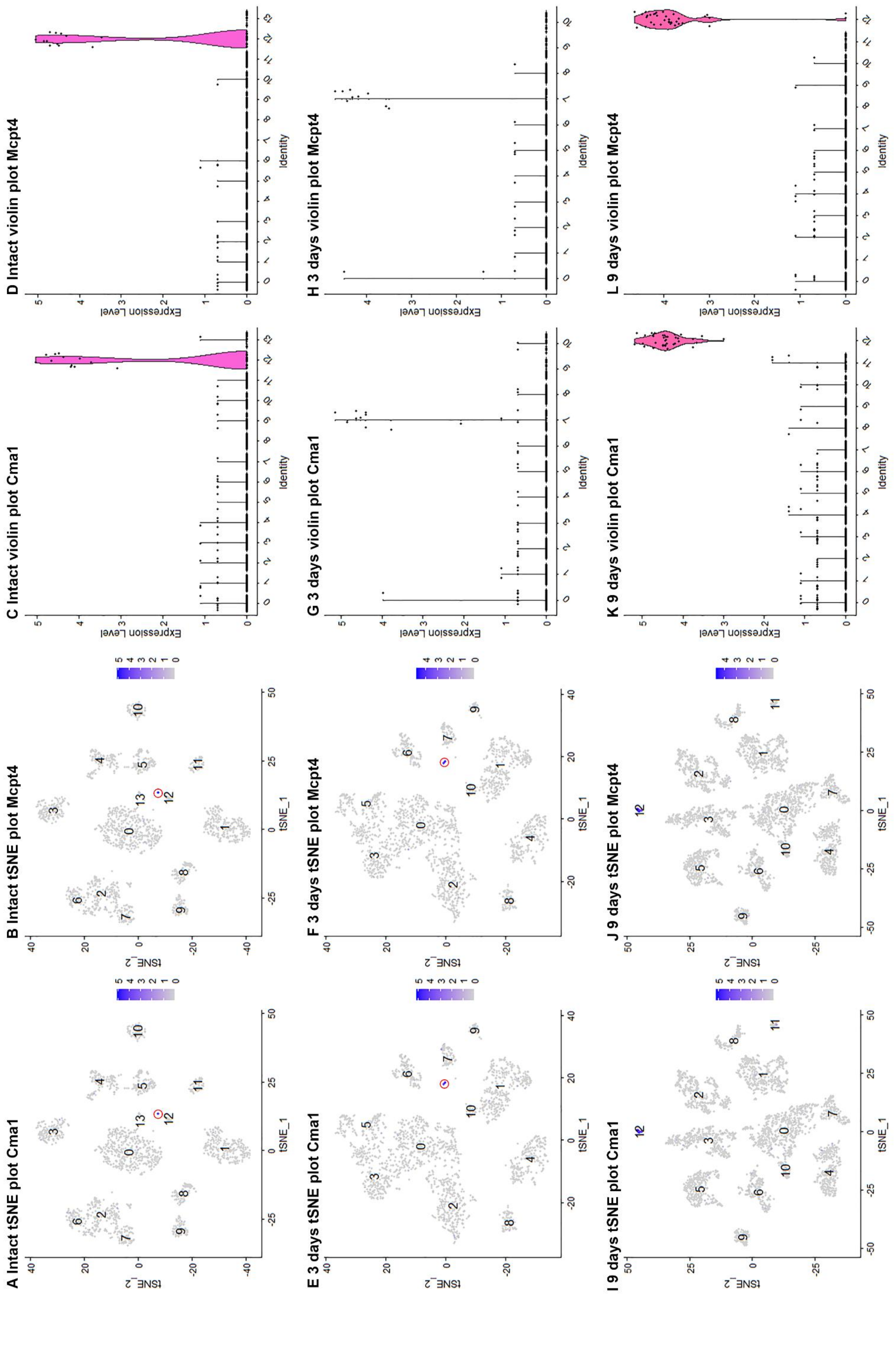
A Intact tSNE plot Cma1
B Intact tSNE plot Mcpt4
C Intact violin plot Cma1
D Intact violin plot Mcpt4
E 3 days tSNE plot Cma1
F 3 days tSNE plot Mcpt4
G 3 days violin plot Cma1
H 3 days violin plot Mcpt4
I 9 days tSNE plot Cma1
J 9 days tSNE plot Mcpt4
K 9 days violin plot Cma1
L 9 days violin plot Mcpt4
tSNE_1
tSNE_2
Expression Level
Identity

Figure S15

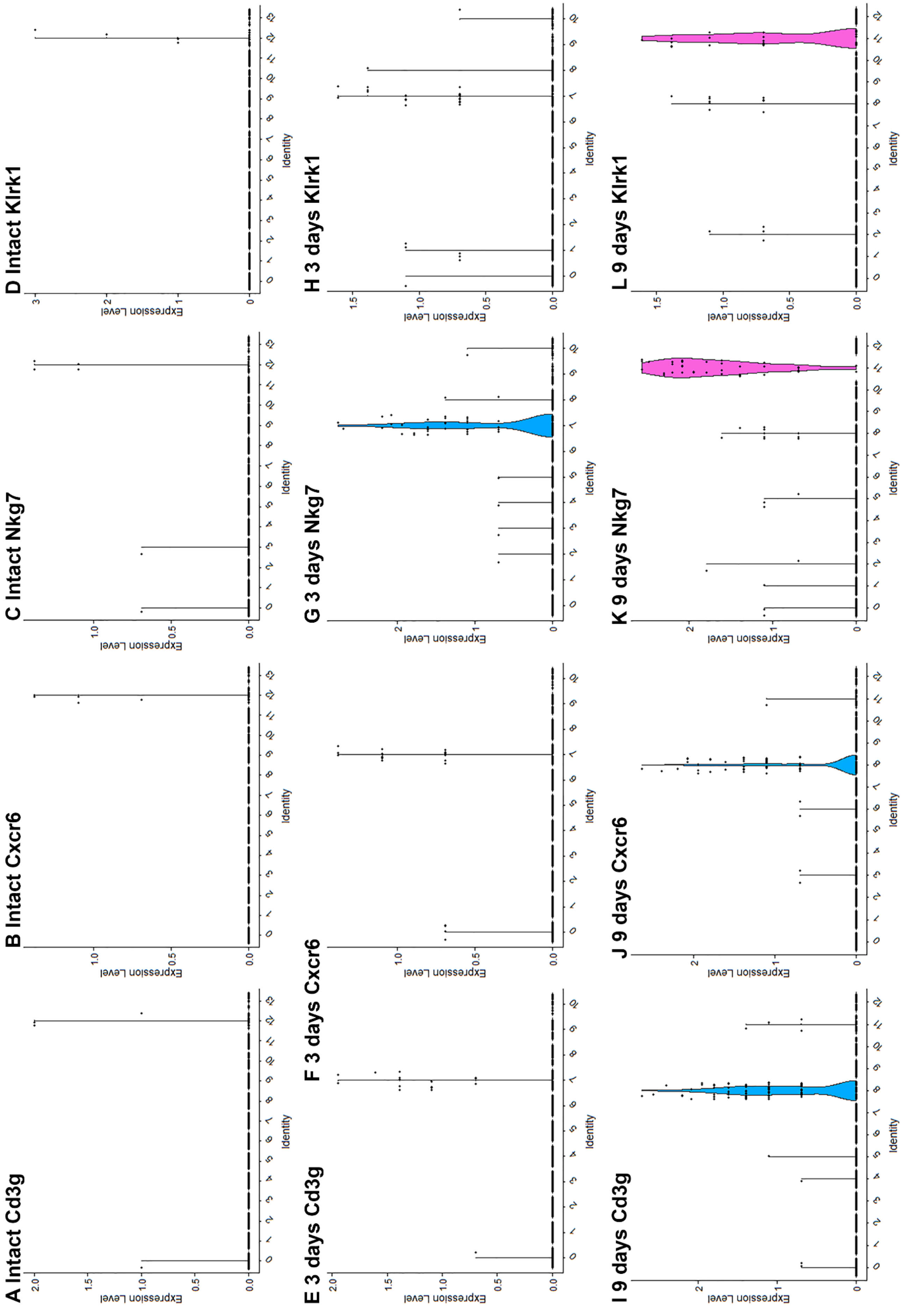
A Intact Cd3g
B Intact Cxcr6
C Intact Nkg7
D Intact Klrk1
E 3 days Cd3g
F 3 days Cxcr6
G 3 days Nkg7
H 3 days Klrk1
I 9 days Cd3g
J 9 days Cxcr6
K 9 days Nkg7
L 9 days Klrk1
Expression Level
Identity

Figure S16

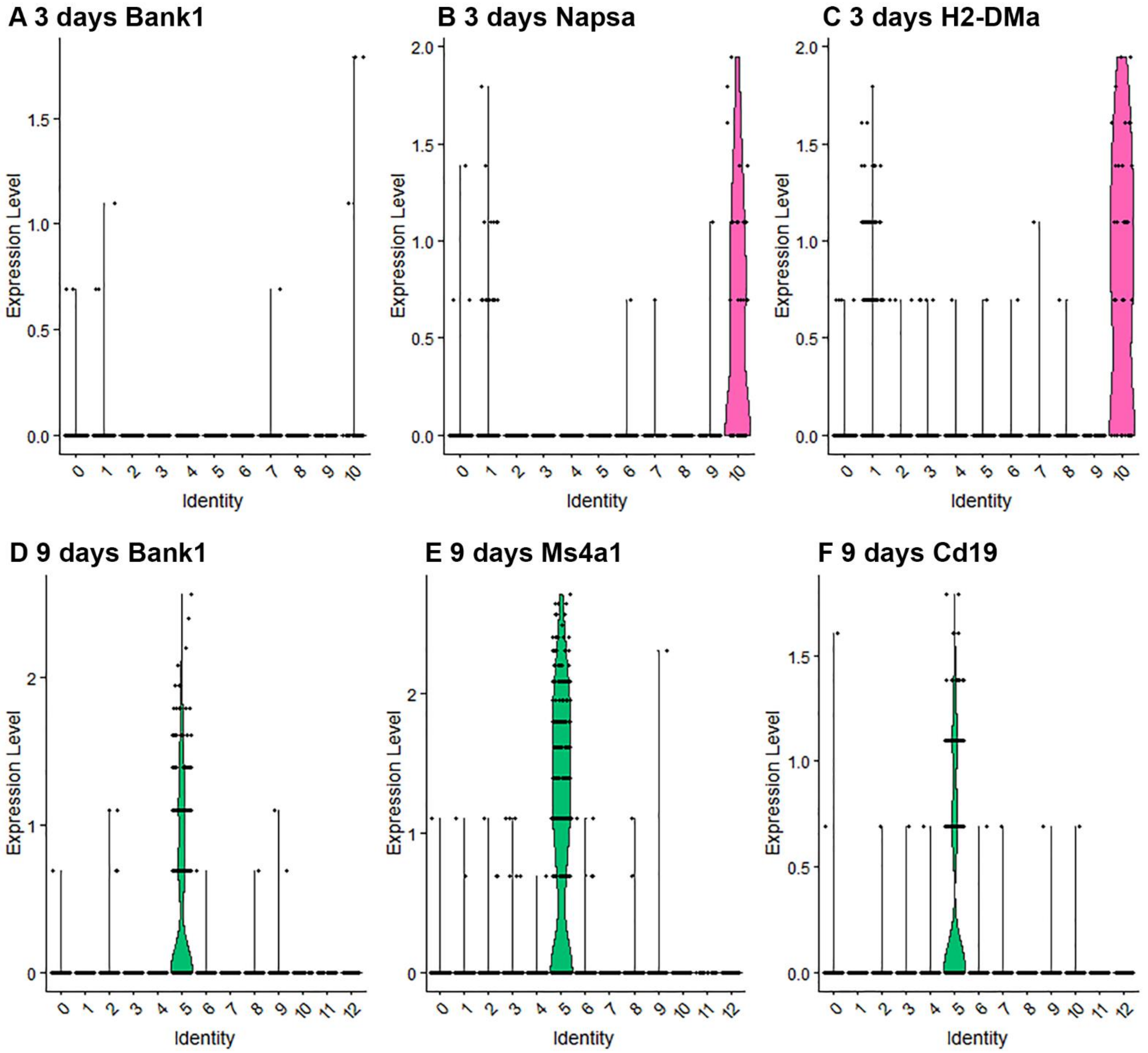

A 3 days Bank1
B 3 days Napsa
C 3 days H2-DMa
D 9 days Bank1
E 9 days Ms4a1
F 9 days Cd19
Expression Level
Identity
0.0
0.5
1.0
1.5
2.0
0
1
2
3
4
5
6
7
8
9
10
11
12

Figure S17

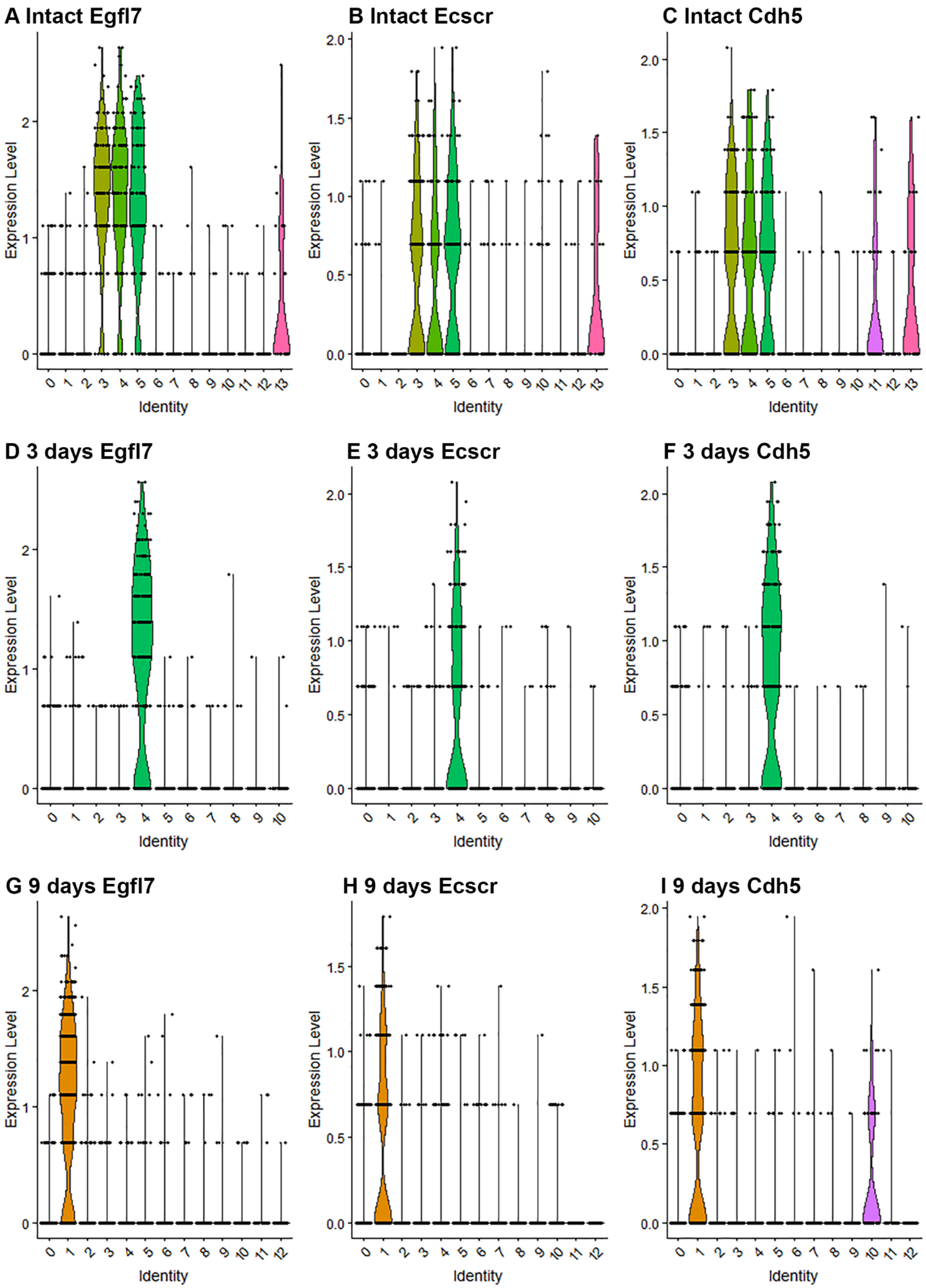
A Intact Egfl7
B Intact Ecscr
C Intact Cdh5
D 3 days Egfl7
E 3 days Ecscr
F 3 days Cdh5
G 9 days Egfl7
H 9 days Ecscr
I 9 days Cdh5
Expression Level
Identity

Figure S1 Violin plot of selected marker genes Lyve1 ($p<0.0001$), Mmrn1 ($p<0.0001$) and Flt4 ($p<0.0001$) for lymphatic endothelial cells. (A) Intact Lyve, (B) Intact Mmrn1, (C) Intact Flt4, (D) 3 days post-injury Lyve, (E) 3 days post-injury Mmrn1, (F) 3 days post-injury Flt4, (G) 9 days post-injury Lyve, (H) 9 days post-injury Mmrn1, (I) 9 days post-injury Flt4.

Figure S2 Violin plot of selected marker genes Pecam1 ($p<0.0001$), Tie1 ($p<0.0001$) and Emcn ($p<0.0001$) for endothelial cells. (A) Intact Pecam1, (B) Intact Tie1, (C) Intact Emcn, (D) 3 days post-injury Pecam1, (E) 3 days post-injury Tie1, (F) 3 days post-injury Emcn, (G) 9 days post-injury, (H) 9 days post-injury Tie1, (I) 9 days post-injury Emcn.

Figure S3 Violin plot of Pdgfra ($p<0.0001$), Ntn4 ($p<0.0001$) and Ntng1 ($p<0.0001$). (A) Intact Pdgfra, (B) Intact Ntn4, (C) Intact Ntng1, (D) 3 days post-injury Pdgfra, (E) 3 days post-injury Ntn4, (F) 3 days post-injury Ntng1, (G) 9 days post-injury Pdgfra, (H) 9 days post-injury Ntn4, (I) 9 days post-injury Ntng1.

Figure S4 Violin plot of selected marker genes Dlk1 ($p<0.0001$), Mest ($p<0.0001$), Cilp ($p<0.0001$) and Tnc ($p<0.0001$) for differentiating fibroblasts. (A) 3 days Dlk1, (B) 3 days Mest, (C) 3 days Cilp, (D) 3 days Tnc, (E) 9 days Dlk1, (F) 9 days Mest, (G) 9 days Cilp, (H) 9 days Tnc.

Figure S5 Violin plot of selected marker genes Ptprc ($p<0.0001$) and Cd52 ($p<0.0001$) for all immune cells. (A) Intact Ptprc, (B) 3 days Ptprc, (C) 9 days Ptprc, (D) Intact Cd52, (E) 3 days Cd52, (F) 9 days Cd52.

Figure S6 Violin plot of selected marker genes Des ($p<0.0001$) and Myh11 ($p<0.0001$) for vasculature-associated smooth muscle (VSM) cells, and Rgs5 ($p<0.0001$) and Kcnj8 ($p<0.0001$) for pericytes. (A) Intact Des, (B) Intact Myh11, (C) Intact Rgs5, (D) Intact Kcnj8, (E) 3 days Des, (F) 3 days Myh11, (G) 3 days Rgs5, (H) 3 days Kcnj8, (I) 9 days Des, (J) 9 days Myh11, (K) 9 days Rgs5, (L) 9 days Kcnj8.

Figure S7 Violin plot of Erbb3 ($p<0.0001$), S100b ($p<0.0001$) and Ngfr ($p<0.0001$). (A) Intact Erbb3, (B) Intact S100b, (C) Intact Ngfr, (D) 3 days post-injury Erbb3, (E) 3 days post-injury S100b, (F) 3 days post-injury Ngfr, (G) 9 days post-injury Erbb3, (H) 9 days post-injury S100b, (I) 9 days post-injury Ngfr.

Figure S8 Violin plot of selected marker genes Sox10 ($p<0.0001$) and Plp1 ($p<0.0001$) for Schwann cells. (A) Intact Sox10, (B) 3 days post-injury Sox10, (C) 9 days post-injury Sox10, (D) Intact Plp1, (E) 3 days post-injury Plp1, (F) 9 days post-injury Plp1, (G) Intact Mbp ($p<0.0001$), (H) Intact mag ($p<0.0001$), (I) Intact Cdh2 ($p<0.0001$), (J) Intact Mpz ($p<0.0001$), (K) Intact Egr2 ($p<0.0001$), (L) Intact L1cam ($p<0.0001$).

Figure S9 Violin plot for Smoc2 and Apod. Smoc2 and Apod are highly expressed in endoneurial fibroblasts and they are not marker genes for nmSCs. (A) Intact Smoc2, (B) Intact Apod, (C) 3 days post-injury Smoc2, (D) 3 days post-injury Apod, (E) 9 days post-injury Smoc2, (F) 9 days post-injury Apod.

Figure S10 (A-E) Violin plot of selected marker genes Mfap5 ($p<0.0001$) and Serpinf1 ($p<0.0001$) for fibroblasts. (A) Intact Mfap5, (B) 3 days post-injury Mfap5, (C) 9 days post-injury Mfap5, (D) Intact Serpinf1, (E) 3 days post-injury Serpinf1, (F) 9 days post-injury Serpinf1. (G-L) Violin plot of selected marker genes Sfrp2 ($p<0.0001$) and Dpt ($p<0.0001$) for epineurial fibroblasts, (G) Intact Sfrp2, (H) 3 days post-injury Sfrp2, (I) 9 days post-injury Sfrp2, (J) Intact Dpt, (K) 3 days post-injury Dpt, (L) 9 days post-injury Dpt.

Figure S11 Violin plot of selected marker genes for endoneurial fibroblasts Sox9 ($p<0.0001$) and Osr2 ($p<0.0001$), and perineurial cells Dkk3 ($p<0.0001$) and Ntn1 ($p<0.0001$). (A) Intact Sox9, (B) 3 days Sox9, (C) 9 days Sox9, (D) Intact Osr2, (E) 3 days Osr2, (F) 9 days Osr2, (G) Intact Ntng1, (H) 3 days Ntng1, (I) 9 days Ntng1, (J) Intact Ntn1, (K) 3 days Ntn1, (L) 9 days Ntn1.

Figure S12 Violin plot of selected marker genes Aif1 ($p<0.0001$), Cd68 ($p<0.0001$) and Mrc1 ($p<0.0001$) for macrophages. (A) Intact Aif1, (B) 3 days Aif1, (C) 9 days Aif1, (D) Intact Cd68, (E) 3 days Cd68, (F) 9 days Cd68, (G) Intact Mrc1, (H) 3 days Mrc1, (I) 9 days Mrc1.

Figure S13 Violin plot of selected marker genes Lyz2 ($p<0.0001$) and Ccl6 ($p<0.0001$) for myeloid cells, Ptprcap ($p<0.0001$) and Trbc2 ($p<0.0001$) for Lymphoid cells. (A) Intact Lyz2, (B) Intact Ccl6, (C) Intact Ptprcap, (D) Intact Trbc2, (E) 3 days post-injury Lyz2, (F) 3 days post-injury Ccl6, (G) 3 days post-injury Ptprcap, (H) 3 days post-injury Trbc2, (I) 9 days post-injury Lyz2, (J) 9 days post-injury Ccl6, (K) 9 days post-injury Ptprcap, (L) 9 days post-injury Trbc2.

Figure S14 tSNE and Violin plot of selected marker genes Cma1 ($p<0.0001$) and Mcpt4 ($p<0.0001$) for mast cells. (A) Intact tSNE plot Cma1, red circle indicates mast cells. (B) Intact tSNE plot Mcpt4, red circle indicates mast cells. (C) Intact violin plot Cma1, (D) Intact violin plot Mcpt4, (E) 3 days post-injury tSNE plot Cma1, red circle indicates mast cells. (F) 3 days post-injury tSNE plot Mcpt4, red circle indicates mast cells. (G) 3 days post-injury violin plot Cma1, (H) 3 days post-injury violin plot Mcpt4, (I) 9 days post-injury tSNE plot Cma1, (J) 9 days post-injury tSNE plot Mcpt4, (K) 9 days post-injury violin plot Cma1, (L) 9 days post-injury violin plot Mcpt4days Ms4a1.

Figure S15 Violin plot of selected marker genes Cd3g ($p<0.0001$) and Cxcr6 ($p<0.0001$) for T cells, Nkg7 ($p<0.0001$) and Klrk1 ($p<0.0001$) for natural killer (NK) cells. (A) Intact Cd3g, (B) Intact Cxcr6, (C) Intact Nkg7, (D) Intact Klrk1, (E) 3 days post-injury Cd3g, (F) 3 days post-injury Cxcr6, (G) 3 days post-injury Nkg7, (H) 3 days post-injury Klrk1, (I) 9 days post-injury Cd3g, (J) 9 days post-injury Cxcr6, (K) 9 days post-injury Nkg7, (L) 9 days post-injury Klrk1.

Figure S16 Violin plot of selected marker genes Bank1 ($p<0.0001$), Cbfa2t3 ($p<0.0001$), Taok3 ($p<0.0001$), Ms4a1 ($p<0.0001$) and Cd19 ($p<0.0001$) for B cells. (A) 3 days post-injury Bank1, (B) 3 days post-injury Cbfa2t3, (C) 3 days post-injury Ms4a1, (D) 9 days post-injury Bank1, (E) 9 days post-injury Ms4a1, (F) 9 days post-injury Cd19.

Figure S17 Violin plot of selected marker genes Egfl7 ($p<0.0001$), Ecscr ($p<0.0001$) and Cdh5 ($p<0.0001$) for endothelial cells. (A) Intact Egfl7, (B) Intact Ecscr, (C) Intact Cdh5, (D) 3 days post-injury Egfl7, (E) 3 days post-injury Ecscr, (F) 3 days post-injury Cdh5, (G) 9 days post-injury Egfl7, (H) 9 days post-injury Ecscr, (I) 9 days post-injury Cdh5.
